# Serotonin and beyond—a tribute to Manfred Göthert (1939-2019)

**DOI:** 10.1007/s00210-021-02083-5

**Published:** 2021-05-15

**Authors:** H. Bönisch, K. B. Fink, B. Malinowska, G. J. Molderings, E. Schlicker

**Affiliations:** 1 Institute of Pharmacology and Toxicology, University of Bonn, Venusberg-Campus 1, 53105 Bonn, Germany; 2 Merz Pharmaceuticals, Frankfurt/Main, Germany; 3 Department of Physiology and Pathophysiology, Medical University of Białystok, Białystok, Poland; 4 Institute of Human Genetics, University of Bonn, Bonn, Germany

**Keywords:** Presynaptic receptors, 5-HT receptor mutants, 5-HT_3_ receptor structure and function, Mode of action of ethanol, Mode of action of anesthetics, Mode of action of gabapentinoids, nACh receptors, NMDA receptors, AMPA receptors, Voltage-dependent cation channels

## Abstract

Manfred Göthert, who had served *Naunyn-Schmiedeberg’s Arch Pharmacol* as Managing Editor from 1998 to 2005, deceased in June 2019. His scientific oeuvre encompasses more than 20 types of presynaptic receptors, mostly on serotoninergic and noradrenergic neurones. He was the first to identify presynaptic receptors for somatostatin and ACTH and described many presynaptic receptors, known from animal preparations, also in human tissue. In particular, he elucidated the pharmacology of presynaptic 5-HT receptors. A second field of interest included ligand-gated and voltage-dependent channels. The negative allosteric effect of anesthetics at peripheral nACh receptors is relevant for the peripheral clinical effects of these drugs and modified the Meyer-Overton hypothesis. The negative allosteric effect of ethanol at NMDA receptors in human brain tissue occurred at concentrations found in the range of clinical ethanol intoxication. Moreover, the inhibitory effect of gabapentinoids on P/Q Ca^2+^ channels and the subsequent decrease in AMPA-induced noradrenaline release may contribute to their clinical effect. Another ligand-gated ion channel, the 5-HT_3_ receptor, attracted the interest of Manfred Göthert from the whole animal via isolated preparations down to the cellular level. He contributed to that molecular study in which 5-HT_3_ receptor subtypes were disclosed. Finally, he found altered pharmacological properties of 5-HT receptor variants like the Arg219Leu 5-HT_1A_ receptor (which was also shown to be associated with major depression) and the Phe124Cys 5-HT_1B_ receptor (which may be related to sumatriptan-induced vasospasm). Manfred Göthert was a brilliant scientist and his papers have a major impact on today’s pharmacology.

On June 28, 2019, Professor Manfred Göthert, former Managing Editor of *Naunyn-Schmiedeberg’s Arch Pharmacol*, passed away in Hamburg, at the age of 79 years. Manfred Göthert was born in 1939 in Braunschweig. He started to study medicine at the University of Hamburg in 1959 and continued studies in Freiburg, Innsbruck, Vienna, and finally Göttingen where he graduated in 1965. In Göttingen, he also prepared his doctoral thesis and received his MD title (Dr. med.) in 1965. In 1967, he joined the Institute of Pharmacology of the University of Hamburg as a postdoctoral scholar where he completed his habilitation thesis in 1971 and received the title “Professor” in 1976. He was called to the University of Essen in 1978 (C3 Professor) and to the University of Bonn in 1985 (C4 Professor) where he was Head of the Institute of Pharmacology and Toxicology until his retirement in 2006. Manfred Göthert served *Naunyn-Schmiedeberg’s Archives of Pharmacology* as editor 1987-1995 and 2002-2003 and as Managing Editor 1995-2002. During his time as Managing Editor jointly with Karl Heinz Jakobs (Aktories et al. 2019), he guided the journal very well and initiated its change from a journal mainly recognized in the German Society for Experimental and Clinical Pharmacology and Toxicology to an internationally recognized platform to publish studies in experimental pharmacology. *Naunyn-Schmiedeberg’s Archives of Pharmacology* is extremely grateful to Manfred Göthert for his long-lasting service and will dearly miss his input.

The scientific work by Manfred Göthert encompasses 271 articles covered in *pubmed*, which appeared during the time period from 1968 (Göthert et al. 1968) to 2020 (Baranowska-Kuczko et al. 2020; Göthert et al. 2020). Since the titles of no less than 110 articles contain *serotonin*, *5-hydroxytryptamine*, or *5-HT* (encompassing eight 5-HT receptors; Tables 1 and 2), the title of this review and the headings of its chapters (“Serotonin” and “Beyond serotonin”) were chosen accordingly.

**Table 1:** Metabotropic 5-HT receptor subtypes examined by Manfred Göthert

^1^Subtype	Function	See
h5-HT_1A_	Unchanged pharmacology of the Ile28Val variant of the human 5-HT_1A_ receptor (Brüss et al. 1995)	“Molecular vistas” section
	Impairment of signal transduction in the Arg219Leu variant of the human 5-HT_1A_ receptor (Brüss et al. 2005a)	“Molecular vistas” section
	Major depression associated with the Arg219Leu variant of the human 5-HT_1A_ receptor gene (Haenisch et al. 2009)	“Molecular vistas” section
r5-HT_1B_	Inhibitory presynaptic autoreceptor and heteroreceptor in rat brain (Engel et al. 1986) and vena cava (Molderings et al. 1987)	“Presynaptic autoreceptors and heteroreceptors” section
h5-HT_1B_	Inhibitory presynaptic autoreceptor in human brain (Schlicker et al. 1997a)	“Presynaptic autoreceptors and heteroreceptors” section
	Postsynaptic receptor involved in the contraction of human temporal arteries (Verheggen et al. 2006)	“Molecular vistas” section
	Reduced surface expression of the Phe124Cys variant of the human 5-HT_1B_ receptor (Brüss et al. 1999b)	“Molecular vistas” section
	The Phe124Cys variant of the human 5-HT_1B_ receptor shows much lower agonist efficacy (Kiel et al. 2000)	“Molecular vistas” section
	Potential role of the Phe124Cys variant in human temporal arteries (Verheggen et al. 2006)	“Molecular vistas” section
h5-HT_1D_	Inhibitory presynaptic heteroreceptor in human atrium (Molderings et al. 1996a)	“Presynaptic autoreceptors and heteroreceptors” section
5-HT_2A_	Postsynaptic receptor involved in rat vascular contraction in vitro (Baumgarten et al. 1972b) and in situ (Göthert et al. 1973)	“Early studies” section
	Postsynaptic receptor involved in the contraction of human temporal arteries in vitro (Verheggen et al. 2006)	“Molecular vistas” section
	Postsynaptic receptor involved in tachycardia in rats in situ (Göthert et al. 1986b)	Not discussed in the text
h5-HT_2C_	Inverse agonist-induced resensitization is more rapid at the Cys23Ser variant than at the wild type (Walstab et al. 2011)	“Molecular vistas” section
5-HT_4_	Facilitatory presynaptic heteroreceptor in rabbit pulmonary artery (Molderings et al. 2006)	“Presynaptic autoreceptors and heteroreceptors” section
5-HT_4_ ?^2^	Inhibitory presynaptic heteroreceptor in pig coronary artery (Molderings et al. 1989a)	“Presynaptic autoreceptors and heteroreceptors” section
h5-HT_7A_	Agonists show lower efficacy and potency at the Pro279Leu variant than at the wild-type receptor (Kiel et al. 2003)	“Molecular vistas” section
	Agonists show much lower affinity to the Thr92Lys receptor variant (Brüss et al. 2005b)	“Molecular vistas” section

^1^*h* and *r* designate human and rat, respectively. This is e.g. interesting for the 5-HT_1B_ and 5-HT_1D_ receptors since the species homologs r5-HT_1B_ and h5-HT_1B_ receptors markedly differ in their pharmacological properties, whereas the h5-HT_1B_ and h5-HT_1D_ receptors (former designations: 5-HT_1Dß_ and 5-HT_1Dα_, respectively) are very similar in this respect

^2^The pharmacological properties most closely fit to the 5-HT_4_ receptor, which is, however, not a likely candidate since 5-HT_4_ receptors are linked to G_s_ protein, whereas inhibitory presynaptic receptors are usually G_i/o_ protein-linked

**Table 2:** 5-HT_3_ receptors examined by Manfred Göthert

	See
*Function in whole animal*	
Receptor involved in the emetogenic effect of cisplatin in domestic pig (Szelenyi et al. 1994)	Not discussed in the text
Cocaine-induced hyperlocomotion of rats (Przegaliński et al. 2005)	“Cannabinoids” section
5-HT-induced activation of Bezold-Jarisch reflex in rats (Malinowska et al. 1995, 1996)	“Cannabinoids” section
*Function in isolated tissue*	
5-HT-induced facilitation of catecholamine release in bovine adrenal medulla (Göthert et al. 1976a)	“Ethanol, general anesthetics, gabapentinoids, and ion channels” section
5-HT-induced facilitation of noradrenaline release via presynaptic heteroreceptors in rabbit heart (Göthert and Dührsen 1979; Göthert and Thielecke 1976)	“Early studies” and “Ethanol, general anesthetics, gabapentinoids, and ion channels” sections
*Tools and studies at the cellular, subcellular, or molecular level*	
Agonist-induced ^*14*^*C-guanidinium influx* through the 5-HT_3_ receptor channel of mouse N1E-115 cells: basic pharmacology (Bönisch et al. 1993)	“Molecular vistas” section
5-HT-induced ^14^C-guanidinium influx through the 5-HT_3_ receptor channel of mouse N1E-115 cells: effect of anesthetics (Barann et al. 1993)	“Molecular vistas” section
5-HT-induced ^14^C-guanidinium influx through the 5-HT_3_ receptor channel of mouse N1E-115 cells: effect of alcohols and substance P (Barann et al. 1995)	“Molecular vistas” section
5-HT-induced ^14^C-guanidinium influx through the 5-HT_3_ receptor channel of mouse N1E-115 cells: effect of steroids (Barann et al. 1999)	“Molecular vistas” section
5-HT-induced ^14^C-guanidinium influx and ^*3*^*H-GR65630 binding* in N1E-115 cells: effect of replacement of sodium ions (Barann et al. 2004)	“Molecular vistas” section
*Patch-clamp studies* at mouse 5-HT_3_ receptors of N1E-115 cells: basic properties and effects of pentobarbital (Barann et al. 1997)	“Molecular vistas” section
Patch-clamp studies at mouse 5-HT_3_ receptors of N1E-115 cells: effect of ifenprodil (Barann et al. 1998)	“Molecular vistas” section
Cloning and functional analysis (in transfected cells) of the human 5-HT_3_ receptor and of two splice variants (Brüss et al. 1998)	“Molecular vistas” section
Patch-clamp and radioligand binding study in transfected HEK293 cells expressing a short splice variant of the mouse 5-HT_3_ receptor (Brüss et al. 1999a)	“Molecular vistas” section
Exon-intron organization of the human 5-HT_3A_ receptor gene (Brüss et al. 2000a)	“Molecular vistas” section
Modification of 5-HT_3_ receptor function by co-expression of alternatively spliced isoforms (Brüss et al. 2000b)	“Molecular vistas” section
Patch-clamp studies in patches from HEK293 cells transfected with the cDNA of the human 5-HT_3_ receptor: effect of barbiturates (Barann et al. 2000)	“Molecular vistas” section
Patch-clamp and radioligand binding study in transfected HEK293 cells expressing the Pro391Arg variant of the human 5-HT_3_ receptor (Kurzwelly et al. 2004)	“Molecular vistas” section
Patch-clamp and radioligand binding study in transfected HEK293 cells expressing the Arg344His variant of the human 5-HT_3_ receptor (Combrink et al. 2009)	“Molecular vistas” section
*Aequorin luminescence-based Ca*^*2+*^ *assay* to characterize 5-HT_3_ receptors: establishment of the assay (Walstab et al. 2007)	“Molecular vistas” section
Characterization of the novel human receptor subunits 5-HT_3C_, 5-HT_3D_ and 5-HT_3E_ (Niesler et al. 2007)	“Molecular vistas” section

## Serotonin

Manfred Göthert published his first papers dedicated to serotonin in 1972 and his interest in this monoamine lasted up to his death. His scientific activities directed towards serotonin may be differentiated into three periods (as reflected by the headings “Early studies”, “Presynaptic autoreceptors and heteroreceptors” and “Molecular vistas”) and roughly correspond to his time spent in Hamburg, Essen, and Bonn, respectively.

### Early studies

Manfred Göthert performed some of his early studies on 5-HT in cooperation with the anatomist H.G. Baumgarten, who had described the 5-HT neurotoxins 5,6- and 5,7-dihydroxytryptamine (5,6- and 5,7-DHT) for the first time (for review, see Jonsson 1980). The question was whether the neurotoxins also affect noradrenergic neurones. In rodents, intraperitoneally administered 5,7-DHT destroyed the postganglionic sympathetic neurones with a potency comparable to that of 6-hydroxydopamine, the standard neurotoxin for noradrenergic neurones (Baumgarten et al. 1974), whereas 5,6-DHT showed such an effect at high doses only (Baumgarten et al. 1972a). Subsequently, the complexity of acute effects of 5-HT and its monohydroxylated and dihydroxylated analogs on cardiovascular parameters was studied in vitro, in situ, and in vivo. The compounds act (i) directly by activation of postsynaptic 5-HT receptors (Rs) (Table 1) and/or via effects on the noradrenergic system including (ii) activation of α-adrenoceptors (α-ARs), (iii) an indirect sympathomimetic effect, and/or (iv) activation of facilitatory presynaptic 5-HT-Rs (Baumgarten et al. 1972b; Göthert et al. 1973; Göthert and Klupp 1978). A masterpiece of this early phase is the paper by Göthert and Dührsen (1979) on rabbit atria (Table 2), in which the chronotropic effect and noradrenaline release were quantified. Infusion of 6-hydroxytryptamine (6-HT) led to a gradual increase in heart rate and noradrenaline release, whereas 5-HT itself and 5,7-DHT caused rapid increases in both parameters, followed by a fading down. Reserpine inhibited all effects, the inhibitor of the neuronal noradrenaline transporter, desipramine, selectively counteracted the effects of 6-HT, whereas the Ca^2+^ antagonist verapamil attenuated the effects of 5-HT and 5,7-DHT. The positive chronotropic effect of 5-HT was desensitized by prior exposure to 5-HT itself or 5,7-DHT. The data clearly revealed that mechanisms (iii) and (iv) are involved, respectively.

The facilitatory presynaptic receptor in the study by Göthert and Dührsen (1979) is a 5-HT_3_-R. This type of receptor can be regarded as a hub within the scientific work of Manfred Göthert (Table 2). One of his coworkers, interested in histamine *H*_*3*_-Rs, sometimes informed him about latest results obtained and he repeatedly responded: You mean *5-HT*_*3*_? The 5-HT_3_-R will re-appear in many sections of this review, e.g. under the “Molecular vistas” section where molecular biological properties of this receptor will be considered. The reason why 5-HT_3_-Rs will also be discussed in the second chapter of this review is that they play a role beyond serotonin, i.e. the 5-HT_3_-R is just one example of ligand-gated ion channels (besides nicotinic acetylcholine (nACh) and/or N-methyl-D-aspartate (NMDA)-Rs) which are targeted by ethanol (“Ethanol, general anesthetics, gabapentinoids, and ion channels” section) and cannabinoids (“Cannabinoids” section).

### Presynaptic autoreceptors and heteroreceptors

#### Presynaptic serotonin autoreceptors

**Fig. 1:**
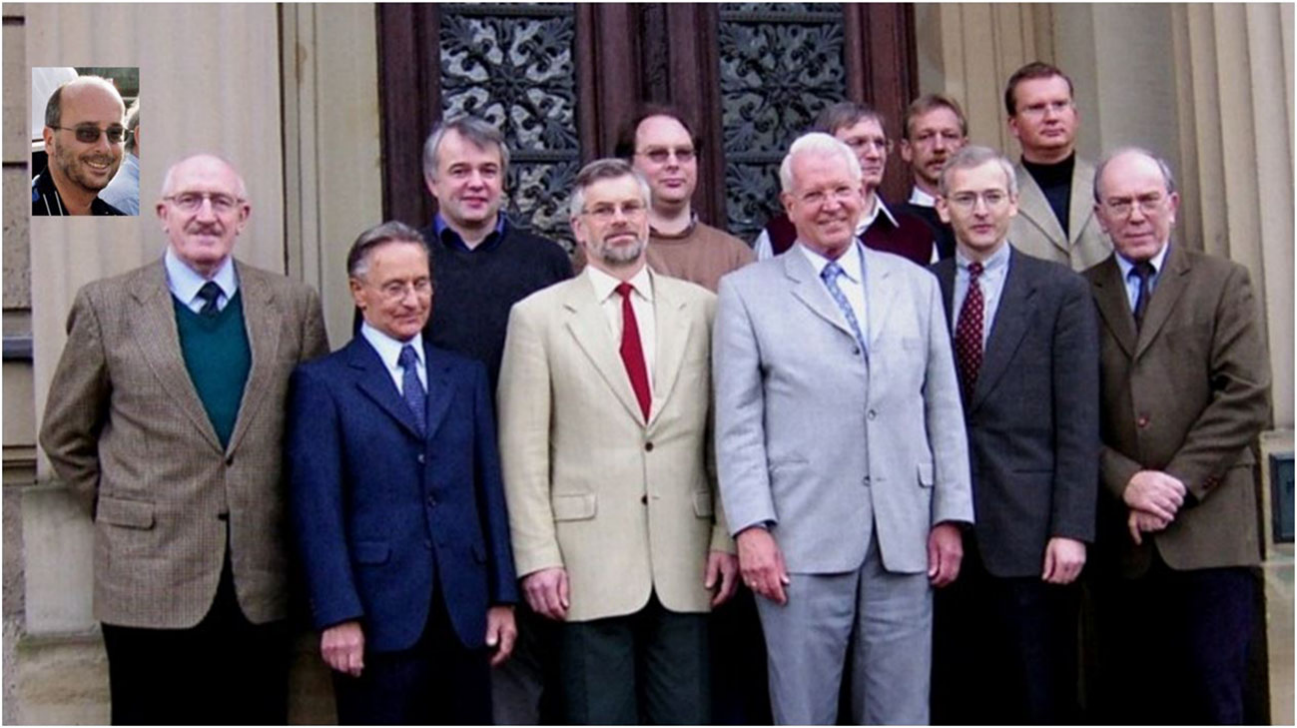
Manfred Göthert and his colleagues of the Institute of Pharmacology and Toxicology, University of Bonn. From left: Martin Barann (inset), Dieter Abbo Kalbhen, Karlfried Karzel, Ivar von Kügelgen, Kurt Racké, Gerhard J. Molderings, Manfred Göthert, Eberhard Schlicker, Michael Brüss, Klaus Fink, Markus Kathmann, and Heinz Bönisch. The photograph was taken on October 29, 2002 in front of the main door of the old institute building in Bonn-Poppelsdorf, Reuterstr. 2b

Presynaptic receptors represent a mechanism by which a transmitter (or a locally formed mediator or a hormone) inhibits or increases the release of the same (autoreceptor) or of another transmitter (heteroreceptor). In 1971, the autoreceptors modulating the release of noradrenaline, acetylcholine, and γ-aminobutyric acid (GABA) from their respective neurones have been described for the first time (reviewed in Starke et al. 1989). It took until 1979 before the *serotonin autoreceptor* was identified, by the groups of Manfred Göthert in Essen (Göthert and Weinheimer 1979) and M. Raiteri in Genova (Cerrito and Raiteri 1979). Both groups examined the depolarization-induced release of tritium from brain preparations preloaded with ^3^H-serotonin. Göthert and Weinheimer (1979) used rat brain cortex slices, whereas Cerrito and Raiteri (1979) performed their study on synaptosomes (i.e., isolated nerve endings) from rat hypothalamus. In subsequent studies, Manfred Göthert, who was supported by E. Schlicker (since 1980) and K. Fink (since 1986) (see Fig. 1), further characterized the 5-HT autoreceptor, particularly the mechanism involved in its action (Göthert 1980a). In addition, a series of drug tools including agonists (Göthert and Schlicker 1983; Göthert et al. 1987; Schlicker et al. 1992a), antagonists (Schlicker and Göthert 1981; Schlicker et al. 1985a), and a 5-HT uptake inhibitor (important for performing superfusion studies; Classen et al. 1984) was examined. The autoreceptor retains its function in spontaneously hypertensive (SHR; Schlicker et al. 1988a) and even in senescent rats (Schlicker et al. 1989a). The 5-HT autoreceptor could also be identified in the human cerebral cortex (Schlicker et al. 1985b) and hippocampus (Schlicker et al. 1996a) and is likely to be involved in the pathogenesis of mood disorders and in the effect of antidepressant drugs (Groß et al. 1987; Starke et al. 1989); it might be a target for antihypertensive drugs (reviewed in Starke et al. 1989).

The major scientific topic in the research on the 5-HT autoreceptor was the determination of the 5-HT subtype. This task proved to be very exciting since 5-HT-R classification was still in its beginning at that time. Gaddum and Picarelli (1957) had proposed D-Rs and M-Rs on the basis of organ bath studies in the guinea-pig ileum. In the seventies, the radioligand binding technique was developed and allowed the rapid determination of receptor affinities of huge amounts of drugs. Peroutka and Snyder (1979) suggested 5-HT_1_-Rs and 5-HT_2_-Rs on the basis of their experiments with ^3^H-5-HT and ^3^H-spiroperidol, respectively. Both nomenclatures show partial overlap only, the D and the 5-HT_2_-R being very similar. To have a unified nomenclature, the D-Rs and M-Rs were re-named 5-HT_2_ and 5-HT_3_, respectively (Bradley et al. 1986).

**Fig. 2:**
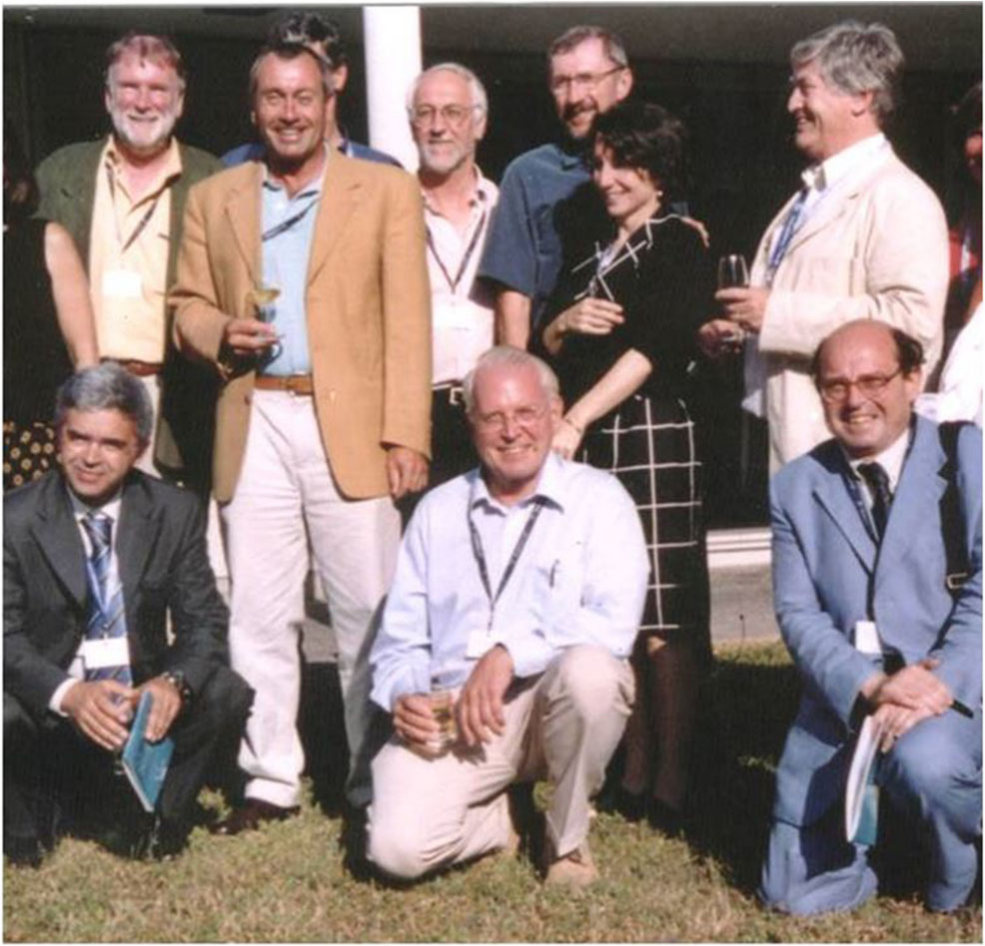
Manfred Göthert and some colleagues. From left, first line: Jorge Gonçalves, Manfred Göthert, and Daniel Moura; second line: Mark Geyer, Daniel Hoyer, Ewan Mylecharane, David Nelson, Stephanie Watts, and Richard Green. The photograph was taken on occasion of the 1st EPHAR Serotonin Satellite Meeting in Porto (Portugal) in July 2004 organized by the International Society for Serotonin Research (formerly The Serotonin Club). Note that Moura (Molderings et al. 1993) and Hoyer (e.g., Engel et al. 1986) have cooperated with Manfred Göthert

To determine the pharmacological properties of the 5-HT autoreceptor in the rat brain, Manfred Göthert cooperated with G. Engel and D. Hoyer from Sandoz (now Novartis) in Basle, who contributed radioligand binding studies (see Fig. 2). Comparison of the potencies of agonists and antagonists at the 5-HT autoreceptor revealed identical properties with their affinities at 5-HT_1_ sites labeled with ^3^H-5-HT as opposed to their potencies at functional 5-HT_2_-Rs and 5-HT_3_-Rs (Engel et al. 1983). Since 5-HT_1_ sites are not homogeneous (Engel et al. 1983), its two components, termed 5-HT_1A_ and 5-HT_1B_, were labeled by ^3^H-8-hydroxy-2-(di-n-propylamino)tetralin (^3^H-8-OH-DPAT) and ^125^I-cyanopindolol (in the presence of isoprenaline), respectively, and the autoreceptor could be sub-classified as 5-HT_1B_ (Engel et al. 1986; Table 1). By the way, the latter article is the most frequently quoted original paper by Manfred Göthert (745 citations; Google Scholar, accessed on March 26, 2021).

Subsequent research revealed that ^3^H-5-HT binds to a third 5-HT_1_-R subtype, termed 5-HT_1D_, in the bovine brain (reviewed by Peroutka 1988). Since the 5-HT_1B_-R was found in the brain of rodents but not of other species (reviewed by Peroutka 1988), the pig brain was chosen as a model for the human brain (Fink et al. 1988). An additional cooperation study with D. Hoyer, based on superfusion and binding studies and a biochemical 5-HT_1D_-R model (inhibition of cAMP formation), revealed that the 5-HT autoreceptor in the pig brain can be classified as 5-HT_1D_-R (Schlicker et al. 1989b; Table 1).

With the advent of molecular biological methods, it became evident that 5-HT_1D_-Rs are heterogeneous; the two sub-subtypes were originally termed 5-HT_1Dα_ and 5-HT_1Dβ_, respectively (Hoyer et al. 1994). Using ketanserin, which has a higher affinity for the former than for the latter receptor (Hoyer et al. 1994), the autoreceptor in not only the guinea-pig cerebral cortex (Bühlen et al. 1996), which served as a model for the human brain, but also in the human cerebral cortex itself could be classified as 5-HT_1Dβ_-R (Fink et al. 1995a; Table 1).

Comparison of the amino acid sequence of the human 5-HT_1Dα_-R and 5-HT_1Dβ_-R revealed an overall identity of 63% only although the pharmacological properties of both receptors are very similar. On the other hand, the amino acid sequence of the human 5-HT_1Dβ_-R shows an overall identity of 93% with that of the rat 5-HT_1B_-R; this is in marked contrast to the pronounced difference in the pharmacological properties (Price et al. 1997). Interesting enough, the exchange of one amino acid (Thr355Asn) conferred the pharmacological properties of the rat 5-HT_1B_ to the human 5-HT_1Dβ_-R (Oksenberg et al. 1992). In other words, the human 5-HT_1Dβ_-R is the species homolog of the rat 5-HT_1B_-R and consequently was re-named h5-HT_1B_-R, whereas the human 5-HT_1Dα_-R was re-termed h5-HT_1D_-R (Hartig et al. 1996). In cooperation with D.N. Middlemiss and G.W. Price from SmithKline Beecham (now GlaxoSmithKline) in Harlow, who contributed two selective h5-HT_1B_-R antagonists (SB-216641, SB-236057) and one selective h5-HT_1D_-R antagonist (BRL-15572), the final proof that the 5-HT autoreceptor in the human (and guinea-pig) cerebral cortex is h5-HT_1B_ was possible (Schlicker et al. 1997a; Middlemiss et al. 1999; Table 1).

The 5-HT_5A_-R, which was described for the first time in 1992/3, is G_i/o_ protein-coupled, like the 5-HT_1_-R subtypes (reviewed in Göthert et al. 2020). The possibility that it may serve as an additional inhibitory 5-HT autoreceptor was considered in a cooperation project with G. Groß from Abbott in Ludwigshafen. However, a 5-HT autoreceptor can be excluded at least for the mouse brain cortex and hippocampus since the highly selective 5-HT_5A_-R antagonist A-763079 did not increase 5-HT release nor did it shift the concentration-response curve of the unselective 5-HT-R agonist 5-CT to the right (Drescher et al. 2007).

#### Presynaptic serotonin heteroreceptors on noradrenergic neurones

The question whether 5-HT-Rs also serve as *heteroreceptors* on noradrenergic neurones has been studied as well and revealed different results in central and peripheral neurones. In rodent brain cortex slices, neither inhibitory (Göthert and Schlicker 1991) nor facilitatory (Schlicker et al. 1994a) 5-HT-Rs could be identified. By contrast, both inhibitory and facilitatory presynaptic 5-HT-Rs could be identified on sympathetic neurones innervating cardiovascular tissues. The facilitatory 5-HT_3_-Rs in the rabbit heart have already been discussed above (Göthert and Dührsen 1979).

Much emphasis was put on inhibitory presynaptic 5-HT-Rs. Although such a receptor had already been described in canine blood vessels (McGrath 1977), several new locations (human atrial appendages and saphenous vein; porcine coronary artery; rabbit pulmonary artery; rat vena cava) have been identified by Manfred Göthert; he was supported in this respect by G.J. Molderings (see Fig. 1) since 1986. The possibility that inhibitory 5-HT-Rs on the sympathetic neurones in the heart and in resistance vessels (identified in the pithed rat preparation; Göthert et al. 1986b) are involved in antihypertensive drugs targeting the 5-HT system had to be considered and again precise determination of the 5-HT-R subtype appeared mandatory. Presynaptic receptors were examined in superfused tissues preloaded with ^3^H-noradrenaline and the potencies of agonists and antagonists were, at least in some of the studies, correlated with their affinities in radioligand studies with native or recombinant 5-HT-Rs. The 5-HT-R in the rat vena cava (Göthert et al. 1986b) could be identified as r5-HT_1B_-R (Molderings et al. 1987; Table 1) and therefore resembles the autoreceptor in the brain of this species (see above Engel et al. 1986). On the other hand, the 5-HT-R in human atrial appendages (Molderings et al. 1996a; Schlicker et al. 1997a) and most probably also its counterpart in the human saphenous vein (Göthert et al. 1986a; Molderings et al. 1990) are h5-HT_1D_-Rs (Table 1); thus, they differ from the central autoreceptor, which is a h5-HT_1B_-R (see above Schlicker et al. 1997a; Middlemiss et al. 1999). One might have expected that the 5-HT-R in the pig coronary artery closely resembles the h5-HT_1D_-R but surprisingly it could not be ascribed to any of the 5-HT_1_-R subtypes and in pharmacological terms most closely resembles the 5-HT_4_-R (Molderings et al. 1989a; Table 1). Finally, the situation is particularly complicated in the rabbit pulmonary artery (Molderings et al. 2006). An inhibitory effect on noradrenaline release occurs indirectly via 5-HT_4_-Rs and directly via 5-HT_1_-Rs (Table 1). The 5-HT_4_-Rs are located presynaptically on cholinergic neurones where they increase acetylcholine release; acetylcholine in turn activates inhibitory muscarinic acetylcholine (mACh)-Rs on the postganglionic sympathetic neurones. By contrast, the inhibitory 5-HT_1B_-Rs or 5-HT_1D_-Rs (subtype not determined) are located on the sympathetic neurones themselves. The latter receptors decrease noradrenaline release in the presence of the mACh-R antagonist atropine only. The likely reason is that mACh activation abrogates the 5-HT_1B/D_-R-mediated effect (an analogous type of receptor interaction has been studied for the α_2_-AR and the 5-HT_1B_-R in the rat vena cava; see next paragraph).

The inhibitory effects mediated via presynaptic 5-HT-Rs were less pronounced than the α_2_-autoreceptor-mediated effects and sometimes were totally missing (human pulmonary artery; Freeman et al. 1981). This phenomenon, which casts some doubt on the physiological relevance of the presynaptic inhibitory 5-HT-Rs, is, however, at least partially related to the experimental conditions. Usually, the electrical stimulation used to evoke quasi-physiological ^3^H-noradrenaline release is extending over a time period of several minutes and for this reason, released noradrenaline can accumulate in the biophase of the axon terminals of the postganglionic sympathetic neurones; this phenomenon is even aggravated since the experiments are carried out in the presence of an inhibitor of noradrenaline re-uptake. Molderings and Göthert (1990) showed in the rat vena cava that the extent of the 5-HT-R-related inhibition of noradrenaline release was attenuated by α_2_-AR agonists and increased by antagonists of this receptor suggesting a receptor interaction between the α_2_-auto- and 5-HT_1B_-heteroreceptor (reviewed in Schlicker and Göthert 1998). Since α_2_-AR agonists and antagonists decrease and increase noradrenaline release, the possibility had to be considered that their modulatory effects on the 5-HT-R-related inhibition are related to their effects on noradrenaline release per se rather than to their effects on the α_2_-ARs. This possibility, however, could be excluded since the alteration of the inhibitory effect of 5-HT also occurred when noradrenaline release was adjusted by modification of the stimulation parameters. The study by Molderings and Göthert (1990) also explains findings in the rat vena cava that the extent of inhibition elicited by the 5-HT_1_-R agonist RU 24969 in the presence of an α-AR antagonist was much higher than the inhibitory effect of 5-HT in its absence (Schlicker et al. 1988d).

#### Presynaptic heteroreceptors on serotoninergic neurones

**Fig. 3:**
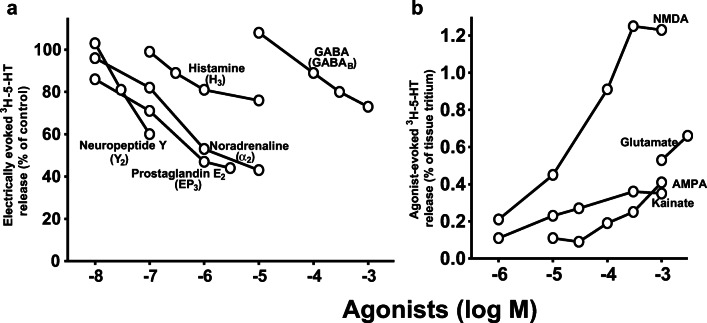
Inhibitory and facilitatory presynaptic heteroreceptors on serotoninergic neurones in rat brain cortex slices identified by Manfred Göthert. **a** The inhibitory effect of five transmitters or mediators leading to *inhibition* of the electrically (3 Hz) evoked ^3^H-5-HT release (the receptors are given in parentheses). The curves were re-drawn from Schlicker et al. (1991)—neuropeptide Y; Schlicker et al. (1987b)—prostaglandin E_2_; Göthert et al. (1983a)—noradrenaline; Schlicker et al. (1988b)—histamine; Schlicker et al. (1984a)—GABA. **b **Glutamate and the prototypical agonists at the three ionotropic glutamate receptors (AMPA, kainate, NMDA) *facilitate* ^3^H-5-HT release. Re-drawn from Fink et al. (1995b). In both panels, SEM values and statistics are not shown. *AMPA*, α-amino-3-hydroxy-5-methyl-4-isoxazolepropionic acid; *GABA*, γ-aminobutyric acid; *NMDA*, N-methyl-D-aspartate

Finally, many efforts were dedicated to the identification of *presynaptic heteroreceptors on the serotoninergic neurones* in the brain. Manfred Göthert has examined inhibitory (Fig. 3a) and facilitatory heteroreceptors (Fig. 3b) in the rat brain cortex. In cortex slices, the excitatory amino acid glutamate evoked 5-HT release; its facilitatory effect was mimicked by agonists of the three types of ionotropic glutamate-Rs. The facilitatory effect of each of them was markedly inhibited by tetrodotoxin (which inhibits propagation of action potentials), suggesting that part of the AMPA-Rs, kainate-Rs, and NMDA-Rs are located presynaptically on the serotoninergic nerve endings (Fink et al. 1995b; Fig. 3b). Inhibitory presynaptic receptors were identified in slices and/or synaptosomes in which depolarization-induced 5-HT release was studied (Fig. 3a); the presynaptic receptors for histamine (Schlicker et al. 1988b), neuropeptide Y (Michel et al. 1990), and prostaglandins of the E series (Schlicker et al. 1987b) were identified for the first time. Although evidence for the existence of α-ARs on serotoninergic neurones has been presented by other authors before (Starke and Montel 1973; Farnebo and Hamberger 1974), final proof came from the study by Göthert and Huth (1980), in which the interaction of noradrenaline with an α-AR antagonist was studied. This receptor (i) belongs to the α_2_-AR subtype (Göthert et al. 1981a); (ii) may be subject to an endogenous tone, i.e. is also activated by endogenous noradrenaline (Göthert and Huth 1980; Schlicker et al. 1982; Feuerstein et al. 1993) although the evidence is not unequivocal (Göthert et al. 1981a; Schlicker et al. 1983); (iii) may be inhibitorily coupled to adenylate cyclase (Schlicker et al. 1987a); and (iv) also occurs in the human brain (shown in cooperation with the group of M. Raiteri; Raiteri et al. 1990). The α_2_-AR-mediated effect was increased when rats had been pretreated with 6-hydroxydopamine (to destroy the noradrenergic neurones) 3 weeks before the experiments and decreased when the animals had received desipramine in the drinking water for 3–4 weeks (Schlicker et al. 1982; Feuerstein et al. 1993). The latter finding might partially explain the delayed effect obtained with antidepressant drugs.

### Molecular vistas

#### Basic properties of ligand-gated 5-HT_3_ receptors

When in 1988 H. Bönisch moved from the University of Würzburg to the University of Bonn (to the Institute of Pharmacology and Toxicology, headed by Manfred Göthert since 1985; see Fig. 1), he introduced two important techniques, which led to a lively collaboration between his group (including M. Brüss and M. Barann) and Manfred Göthert. The culture of human or animal cell lines natively expressing a receptor of interest and, much more important, the establishment of molecular biology methods (such as cDNA cloning, site-directed mutagenesis, transfection of cells, and expression of receptors) enabled studies at the cellular and molecular level.

The first common project with Manfred Göthert was the characterization of 5-HT_3_-Rs in vitro (at the cellular or subcellular level). Altogether we used four different techniques to examine this receptor in rodent cell lines natively expressing the 5-HT_3_-R or in cells transfected with the cDNA of the mouse or human receptor, namely by measuring (i) the 5-HT-induced influx of a radioactively labeled cation through the cation channel of the receptor (Bönisch et al. 1993), (ii) the binding of a radioligand to the receptor protein (Barann et al. 2004), (iii) 5-HT-induced membrane potential changes in patches of cells by means of patch-clamp techniques (Barann et al. 1997), and (iv) aequorin bioluminescence changes caused by the 5-HT-induced Ca^2+^ influx in suspended cells expressing the human 5-HT_3_-R (Walstab et al. 2007). An overview of studies of M. Göthert at 5-HT_3_-Rs is given in Table 2.

Basic *properties of 5-HT*_*3*_*-Rs* were initially examined at N1E-115 mouse neuroblastoma cells which natively express this receptor (Lummis et al. 1990). After Reiser and Hamprecht (1989) had shown that ^14^C-guanidinium is flowing through the open channel of 5-HT_3_-Rs (expressed in neuroblastoma x glioma hybrid cells), Manfred Göthert, in a collaboration with H. Bönisch (and his group), used this method to examine 5-HT_3_-Rs in more detail in mouse neuroblastoma N1E-115 cells (Bönisch et al. 1993). We could show that 5-HT and other 5-HT_3_-R agonists (e.g., phenylbiguanide, 2-methyl-5-HT) cause a concentration-dependent influx of this radioligand which, in contrast to the influx elicited by veratridine, was not inhibited by tetrodotoxin or 5-HT_1_-R, 5-HT_2_-R or 5-HT_4_-R antagonists but inhibited by ondansetron and other selective 5-HT_3_-R antagonists. All examined 5-HT_3_-R agonists caused bell-shaped concentration-response curves with slope factors of the ascending part of about 2, indicating rapid desensitization and positive cooperativity. The 5-HT-induced influx of the organic cation ^14^C-guanidinium was increased in the absence of Ca^2+^ indicating that Ca^2+^ accelerates desensitization kinetics. The 5-HT effect was potentiated by the neurokinin substance P and this potentiation was inhibited by ondansetron. This phenomenon had also been shown before by Reiser and Hamprecht (1989), and later Emerit et al. (1993) could demonstrate that in mouse NG108-15 cells, this potentiating effect was even more pronounced with substance P derivatives which are inactive at the various neurokinin-R classes. We additionally showed that substance P also potentiates the 5-HT_3_-R-mediated Bezold-Jarisch reflex (Malinowska et al. 1996; see the “Cannabinoids” section below and Table 2). In this study, Manfred Göthert concluded that substance P acts at an allosteric modulatory site of the 5-HT_3_-R, thus, producing an increase in cation flux through this channel, e.g. by affecting its open frequency or duration, without necessarily influencing its ligand recognition (orthosteric) site. We later identified ethanol (and other alcohols) as further positive allosteric modulators at 5-HT_3_-Rs of N1E-115 cells (Barann et al. 1995). Ethanol not only increased the 5-HT-induced cation influx (without affecting the 5-HT-induced inhibition of ^3^H-GR65630 binding) but it also abolished the descending part of the concentration-response curve for 5-HT_3_. The potentiating effect of alcohols (n-alkanols) showed the following rank order: methanol < ethanol < n-propanol, i.e., it increased with their lipophilicity. Interestingly, when in the presence of substance P the 5-HT-induced cation influx was already enhanced, the ability of ethanol to increase the 5-HT-induced influx was considerably diminished. Thus, alcohols (n-alkanols) by interacting with a modulatory hydrophobic region of the 5-HT_3_-R may either stabilize the open state or decrease desensitization as proposed by Davies (2011) for further positive allosteric modulators of the 5-HT_3_-R.

In N1E-115 mouse neuroblastoma cells, we studied the influence of sodium ion substitutes on the 5-HT-induced flux of ^14^C-guanidinium through the cation channel of the 5-HT_3_-R and on the competition of 5-HT with binding of the selective 5-HT_3_-R antagonist ^3^H-GR 65630 (Barann et al. 2004). Replacement of sodium by the organic cation choline caused both a rightward shift of the 5-HT concentration-response curve and an increase in the maximum effect of 5-HT, whereas replacement of Na^+^ by Li^+^ had no effect on the potency and maximal response of 5-HT. Replacement by Tris (tris(hydroxymethyl)aminomethane), tetramethylammonium (TMA), or N-methyl-D-glucamine (NMDG) caused an increase in the maximal response to 5-HT similar to that caused by choline. The potency of 5-HT was only slightly reduced by Tris, to a high degree decreased by TMA and choline, but not influenced by NMDG. The potency of 5-HT in inhibiting ^3^H-GR65630 binding to intact cells was much lower when sodium was replaced by choline, but remained unchanged after replacement by NMDG. These results indicate that NMDG, in contrast to choline, is a suitable sodium substituent for studies of 5-HT-evoked ^14^C-guanidinium flux through 5-HT_3_-R channels since it increases the signal-to-noise ratio without interfering with 5-HT binding (Barann et al. 2004). The results also suggest that choline might compete with 5-HT for binding to the 5-HT_3_-R and that the increased maximum response may be partly due to a choline-mediated delay of the 5-HT-induced desensitization.

Using the same techniques and cells, we examined several pharmacologically active compounds for their affinity to this receptor. In N1E-115 cells (and in rat brain cortical membranes), anpirtoline, a highly potent 5-HT_1B_-R agonist, behaved as 5-HT_3_-R antagonist (Göthert et al. 1995a). Both the 5-HT_3_-R channel and the voltage-gated sodium channel of N1E-115 cells were shown to be targets of steroids; however, their interaction is obviously due to a non-specific hydrophobic effect (Barann et al. 1999). Furthermore, imidazolines (e.g., idazoxane, cirazoline, or clonidine) as well as some σ ligands (e.g., ifenprodil) showed low inhibitory potencies at 5-HT_3_-Rs and it was suggested that they may exert their inhibitory effect on cation influx through the 5-HT_3_-R channel, at least in part, by interacting with σ_2_ binding sites (Molderings et al. 1996b).

By the installation of a patch-clamp workstation in his institute, Manfred Göthert initiated the establishment of the patch-clamp technique which finally was introduced by M. Barann. In superfused outside-out patches of N1E-115 cells, we examined further basic properties of the mouse 5-HT_3_-Rs in detail (Barann et al. 1997). We could show that at negative membrane potentials, 5-HT caused concentration-dependent inward currents which were characterized by a Hill coefficient of 1.8 and a peak current of about 21 pA at a high concentration of 5-HT (30 μM). We furthermore demonstrated that the currents induced by 30 μM 5-HT (for 2 s) were characterized by inward rectification, a monophasic onset, and a monophasic decay (desensitization), and that after a short washout period, fully desensitized patches completely recovered (Barann et al. 1997). In this study, we also demonstrated that pentobarbital causes inhibition of the 5-HT_3_-R through an open channel block. 5-HT-induced influx of ^14^C-guanidinium as well as patch-clamp techniques were used to characterize the effects of further anesthetics at the cation channel of the 5-HT_3_-R. By measuring the influx of the organic cation ^14^C-guanidinium induced by either veratridine or 5-HT, the influence of local and general anesthetics on cation influx through the fast, voltage-dependent sodium channel and through the 5-HT_3_-R cation channel was studied in N1E-115 mouse neuroblastoma cells (Barann et al. 1993). The ^14^C-guanidinium influx through both channels was inhibited by local and general anesthetics. With the exception of procaine and cocaine, which were equipotent at both channels, the local anesthetics were 4.4-fold (lidocaine) to 25-fold (tetracaine) more potent at the fast sodium channel than at the 5-HT_3_-R channel. The rank order of potency for general anesthetics was clearly different at the two channels. With the exception of ketamine, which was about equipotent at both channels, the general anesthetics were between 2.2 and 8.1-fold more potent at the 5-HT_3_-R channel than at the fast sodium channel and only at the fast sodium channels, their inhibitory potency was correlated with their lipophilicity. Thus, the relative high inhibitory potencies of the general anesthetics argue in favor of a specific interaction with the 5-HT_3_-R channel (Barann et al. 1993). Using the patch-clamp technique, we re-examined the abovementioned effects of ifenprodil (Molderings et al. 1996b) and we could show that it inhibited the peak currents evoked by 5-HT and that it also produced a concentration-dependent increase of the onset time constant (τ_ON_) of the 5-HT-induced currents and that ifenprodil accelerated current inactivation as reflected by a decrease of the current inactivation time constant (τ_OFF_) (Barann et al. 1998).

#### Molecular biology of 5-HT_3_ receptors

**Fig. 4:**
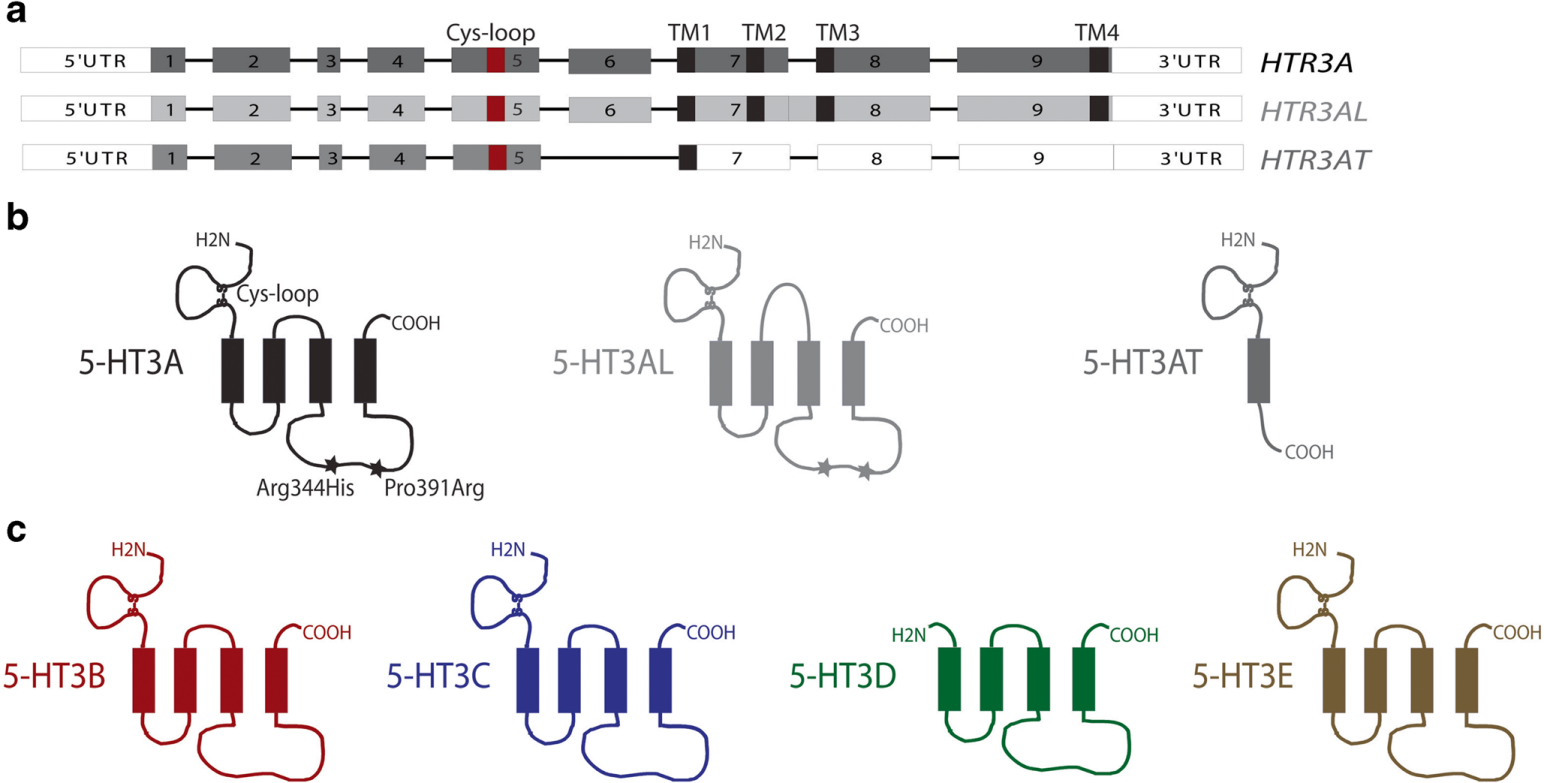
**a** Genomic organization of the human 5-HT_3A_ receptor gene (HTR3A) with exons (indicated by numbers), localization of the Cys-loop (cystein bond within the N-terminal region) and the four transmembrane regions (TM1-TM4), and the organization of the long (HTR3AL) and of the truncated (HTR3AT) splice variant. **b** The corresponding protein structure and the two naturally occurring 5-HT_3A_ receptor variants Arg344His and Pro391Arg due to single-nucleotide polymorphisms of the HTR3A gene. **c **The protein structures of the human 5-HT_3_ receptor subtypes. *UTR*, untranslated region. All these variants and subtypes have been examined by Manfred Göthert (see text)

In 1991, the first findings on the *molecular biology of 5-HT*_*3*_*-Rs* have been published (Maricq et al. 1991). Maricq and coworkers had cloned this receptor from mouse DNA and it was termed as 5-HT_3A_; thereafter, the human 5-HT_3A_ cDNA was cloned by Miyake et al. (1995) and Belelli et al. (1995). All cloned 5-HT_3A_-R cDNAs show a high degree of amino acid identities of more than 80%. Hydrophobicity analysis of the deduced amino acid sequences predicts the receptor subunits to be integral membrane proteins with a large extracellular N-terminus, 4 transmembrane domains (TMs), a large intracellular loop between TM3 and TM4, and a short extracellular C-terminus (see Fig. 4). The ligand-binding domain is proposed at the N-terminal part (containing a Cys-loop, i.e. a cystine pair separated by 13 amino acids, conserved among all ligand-gated ion channels), and TM2 is the putative channel pore-forming domain of this homopentameric ion channel which is closely related to the α-subunit of the nACh-R (Ortells and Lunt 1995). When we cloned the human cDNA of the 5-HT_3A_-R from human amygdala, we amplified three cDNAs of different length, one corresponded to the already known cDNA, whereas the other two were a shorter and a longer alternative splice product (Brüss et al. 1998), and only the longer isoform with an insertion of 96 base pairs leading to the insertion of 32 amino acids into the extracellular loop between TM2 and TM3 was able to form an active receptor protein (Brüss et al. 1998). Both splice variants did not correspond to known mouse isoforms (Jackson and Yakel 1995). We cloned the short splice variant of the mouse 5-HT_3_-R (Hope et al. 1993), expressed the receptor protein in human embryonic kidney (HEK293) cells, and compared its pharmacological properties with those of the native mouse 5-HT_3_-R in N1E-115 neuroblastoma cells by means of ^3^H-GR65630 binding and 5-HT-induced ^14^C-guanidinium influx measurements (Brüss et al. 1999a). The differences between the two isoforms were, however, only marginal and may be due to cell-specific post-translational modifications of the receptor protein in the two cell types (Brüss et al. 1999a). To identify potential alternative exons, we sequenced all exons and introns, the length and positions of all introns of the coding region,and about 19 kb of the 5′-noncoding region of the human 5-HT_3A_-R gene (Brüss et al. 2000a). The human gene stretches over about 14.5 kb. From three published human 5-HT_3A_-R cDNAs, we could confirm only that reported by Miyake et al. (1995); the coding region of the human 5-HT_3A_-R gene is separated by eight introns located at positions nearly identical to those of the murine counterpart (Werner et al. 1994). The length of most introns differs markedly from those of the murine counterpart. Exon 1 encodes the membrane translocation, exons 2 to 6 encode the extracellular N-terminus, exon 7 encodes TM1, TM2, and the extracellular loop between TM2 and TM3, exon 8 codes for TM3, and exon 9 for the large intracellular loop between TM3 and TM4 as well as TM4 and the extracellular C-terminus (see Fig. 4).

In outside-out patches of stably transfected HEK293 cells expressing the recombinant human 5-HT_3A_-R, we characterized basic properties of this receptor and we compared the effects of the barbiturate anesthetics methohexital and pentobarbital (which differ in their lipophilicity) on this receptor channel (Barann et al. 2000). Both anesthetics inhibited the 5-HT response with about equal potency but they clearly differed with respect to the kinetics of their effects indicating that lipophilicity may affect their access to an amphipathic site of action via both a hydrophilic and a hydrophobic pathway.

Of major scientific interest for Manfred Göthert was the exploration of functional consequences of *genetic variations of human 5-HT-Rs*. Concerning the human 5-HT_3A_-R, he was involved in the pharmacological characterization of two naturally occurring variants of the human 5-HT_3A_-R, a Pro391Arg and an Arg344His variant. Both had been detected in schizophrenic patients when the human 5-HT_3A_-R gene was screened for variations in schizophrenic patients and patients suffering from bipolar affective disorder (Niesler et al. 2001). Both missense mutations are located in the second intracellular loop of the receptor protein (see Fig. 4). The variant Pro391Arg receptor was examined in comparison to the wild-type form (each expressed in stably transfected HEK293 cells). In binding experiments with ^3^H-GR65630, the variant receptor exhibited no changes in receptor densities or affinities to diverse 5-HT_3_-R agonists and antagonists, and also the patch-clamp experiments showed no differences between the wild-type and variant receptor (Kurzwelly et al. 2004). Combrink et al. (2009) compared the other receptor variant (Arg344His) in transfected HEK293 cells and in comparison with the wild-type receptor. This comparison was performed using ^3^H-GR65630 binding and patch-clamp analyses including technically demanding single-channel analyses. In addition, 5-HT-induced Ca^2+^ currents through the 5-HT_3A_-R channel were measured by an aequorin luminescence-based Ca^2+^ assay which previously had been established in our group (Walstab et al. 2007). Compared to the wild-type receptor, the density of the variant receptor was decreased by nearly 50%, whereas the Ca^2+^ influx was unchanged. While the radioligand experiments revealed no differences for several agonists and antagonists between wild-type and variant receptor, single-channel analysis suggested an increase in channel open time; this increase appears to compensate for the reduction in variant receptor density.

In 1999, a further 5-HT_3_-R subunit, the 5-HT_3B_-R, was identified (Davies et al. 1999; Dubin et al. 1999), which, in contrast to the 5-HT_3A_-R, is not able to form a functional homopentameric receptor but which was able to cause, when co-expressed with the 5-HT_3A_ subunit, subtle modifications in 5-HT_3_-R agonist and antagonist effects; in addition, heteromeric assemblies of human 5-HT_3A_ and 5-HT_3B_ subunits display larger single-channel conductance than homopentameric assemblies of 5-HT_3A_ subunits (Dubin et al. 1999). Shortly after this report, we described a human short, truncated (5-HT_3AT_) and a long (5-HT_3AL_) splice variant of the human 5-HT_3A_-R subunit (Brüss et al. 2000b). The protein of the short isoform consists of only 238 amino acids with a single transmembrane domain (TM1), whereas the long isoform contains 32 additional amino acids within the extracellular loop between TM2 and TM3 (see Fig. 4). Both splice variants are co-expressed in the amygdala and hippocampus, whereas in the placenta, only the short splice variant is co-expressed (Brüss et al. 2000b). When expressed in transfected HEK293 cells, both splice variants are not able to form a functional receptor, but modify 5-HT responses at heteromeric 5-HT_3A_-Rs. Heteromeric assemblies of 5-HT_3A_ and the 5-HT_3AT_ subunit exhibit much larger 5-HT-induced cation fluxes than homomeric 5-HT_3A_-Rs, whereas heteromeric receptors containing the long splice variant display reduced cation fluxes (Brüss et al. 2000b). Thus, tissue-selective expression of 5-HT_3A_ splice variants may contribute to the functional diversity of this receptor.

Using the aequorin luminescence-based Ca^2+^ assay, which had been shown to be a highly sensitive method for functional characterization of 5-HT_3_-Rs and which allows high-throughput screening (Walstab et al. 2007), we characterized three novel human 5-HT_3_-R subunits, 5-HT_3C_, 5-HT_3D_, and 5-HT_3E_ (Niesler et al. 2007). The proteins of these novel genes, which had been isolated by Niesler et al. (2003), show the following structures: 5-HT_3C_ and 5-HT_3E_ present a huge N-terminal extracellular segment containing a cysteine loop, four hydrophobic TMs, a large intracellular loop between TM3 and TM4, and an extracellular C-terminus (see Fig. 4). The architecture of the 5-HT_3D_ subunit (Fig. 4) is rather different, since it lacks the signal sequence and the large N-terminal region, including the ligand-binding site, indicating that it may not form a functional ion channel. Interestingly, the genes of the 5-HT_3D_ and 5-HT_3E_ subunits are predominantly or even exclusively (5-HT_3E_) expressed in the gastrointestinal tract (Niesler et al. 2003). Using immunofluorescence and immunoprecipitation of recombinantly expressed proteins, we explored whether they are able to form 5-HT_3_-Rs. Radioligand binding experiments and aequorin luminescence-based Ca^2+^ assays were performed to reveal whether they modulate 5-HT_3_-R function. We found that each of the respective candidates coassembled with 5-HT_3A_. The functional experiments revealed that the 5-HT_3C_, 5-HT_3D_, and 5-HT_3E_ subunits alone cannot form functional receptors. Co-expression with 5-HT_3A_, however, results in the formation of functional heteromeric 5-HT_3_-Rs, which exhibit quantitatively different properties compared with homomeric 5-HT_3A_-Rs (Niesler et al. 2007). An excellent review on genetics, molecular biology, physiology, and pharmacology of 5-HT_3_-Rs has been published by Walstab et al. (2010). It should be noted that M. Brüss (together with M. Göthert and H. Bönisch) had an intense collaboration on 5-HT_3_-Rs with B. Niesler (Department of Human Genetics, University of Heidelberg). The PhD student, J. Walstab, involved in this project was later working as postdoc in Niesler’s laboratory when this collaboration between the universities of Bonn and Heidelberg was continued after the sudden and unexpected death of M. Brüss.

#### Variants of metabotropic 5-HT receptors

Within a collaborative research center (SFB 400: Molecular Basis of CNS Disorders) at the University of Bonn, Manfred Göthert was involved in the *characterization of naturally occurring variants of the human metabotropic 5-HT*_*1A*_*-Rs, 5-HT*_*1B*_*-Rs, 5-HT*_*2C*_*-Rs, and 5-HT*_*7*_*-Rs.* Genetic variations in 5-HT-Rs might contribute not only to genetics of diseases but also to changes in pharmacological properties of these receptors (for a short review, see Göthert et al. 1998). Table 1 shows an overview of all studies of M. Göthert at metabotropic 5-HT-Rs.

**Fig. 5:**
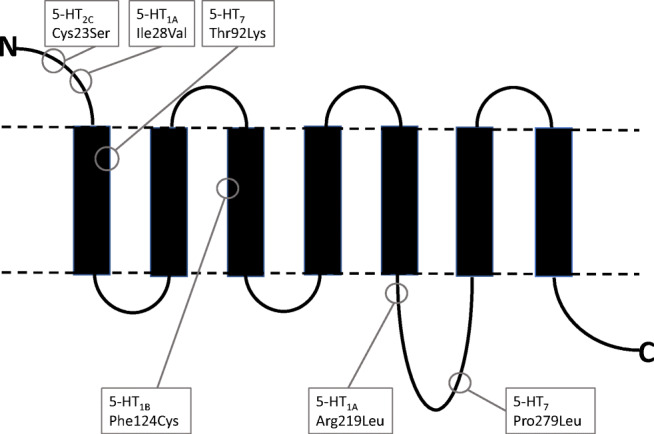
Schematic diagram of a human metabotropic 5-HT receptor in which amino acid exchanges and their position in naturally occurring variants of the 5-HT_1A_, 5-HT_1B_, 5-HT_2C_, and 5-HT_7_ receptor are indicated. Manfred Göthert has explored all shown variants (see text)

Central 5-HT_1A_-Rs act as somadendritic autoreceptors on serotoninergic neurones and in many brain regions, this receptor has been identified in high density, e.g. in the hippocampus and amygdala where it has been assumed to be involved in the regulation of mood and anxiety. This receptor is a target for anxiolytic and antidepressant drugs (Hamon 1997; Blier and De Montigny 1997; Kaufman et al. 2016). 5-HT_1A_-Rs are preferentially coupled to G_i/o_ proteins to inhibit adenylate cyclase but can also be coupled to inwardly rectifying potassium channels mediating hyperpolarization (Andrade and Nicoll 1986; Albert and Vahid-Ansari 2019). In a systematic screening for mutations in the promoter and coding regions of the human 5-HT_1A_ gene, Erdmann et al. (1994) identified two naturally occurring receptor variants with either Ile28Val (in the N-terminus) or Arg219Leu (in the third intracellular loop) substitutions (Table 1, Fig. 5); the allele frequency of the Ile28Val and the Arg219Leu variants is about 8% and <1%, respectively. In transfected COS-7 cells, we could show that the Ile-28-Val exchange had no effect on receptor expression or on the affinities (measured in ^3^H-8-OH-DPAT binding experiments) of a series of agonists or antagonists at 5-HT_1A_-Rs (Brüss et al. 1995). However, the Arg-219-Leu exchange examined later in transfected HEK293 cells by ^3^H-8-OH-DPAT and ^35^S-GTPγS binding (a measure of G protein coupling) to membranes as well as inhibition of forskolin-stimulated ^3^H-cAMP formation by agonists (in whole cells) revealed an impairment of signal transduction (Brüss et al. 2005a). While the variant receptor did not differ from the wild-type receptor with respect to receptor density or potencies of agonists or antagonists, the ability of 5-HT to stimulate ^35^S-GTPγS binding to the variant receptor and of agonists to inhibit forskolin-stimulated cAMP accumulation was decreased by 60-90% (Brüss et al. 2005a). Interestingly, in an association study of major depression with this Arg219Leu variant, we could show that this receptor variant is associated with major depression and that it may play a role in the pathogenesis of depression (Haenisch et al. 2009).

A further naturally occurring 5-HT-R variant examined by Manfred Göthert was the human 5-HT_1B_-R in which Phe in position 124 (within the third transmembrane domain) is substituted by Cys (Table 1, Fig. 5). The allele frequency of this variation is 2%. In transfected COS-7 cells, the Phe124Cys variant, in comparison to the wild-type receptor, showed a reduction by 70% of surface expression (*B*_max_) and two to three times higher affinity for several agonists (e.g., 5-CT or 5-HT) in radioligand binding experiments with ^3^H-5-carboxamidotryptamine (^3^H-5-CT) (Brüss et al. 1999b). This result was confirmed in a second study using transfected C6 glioma cells; this study additionally showed an about 50-65% lower efficacy of agonists (such as 5-CT, 5-HT, or sumatriptan) in stimulating ^35^S-GTPγS binding to membranes of cells expressing the Phe124Cys receptor variant (Kiel et al. 2000). In whole cells expressing the variant receptor, 5-CT and sumatriptan inhibited the forskolin-stimulated cAMP accumulation 3.2-fold more potently than in cells expressing the wild-type receptor. Thus, the Phe-124-Cys mutation modifies the pharmacological properties of the 5-HT_1B_ receptor and may account for pharmacogenetic differences in the action of 5-HT_1B_-R ligands (Kiel et al. 2000).

Manfred Göthert proposed that the sumatriptan-induced vasospasm, which occurs at low incidence as a side effect in migraine therapy, may at least partly be related to the expression of the Phe124Cys variant of the h5-HT_1B_-R in patients with additional pathogenetic factors such as coronary heart disease. This proposal was later tested in human temporal arteries from patients undergoing neurosurgery. These arteries were used to examine whether in vivo expression of the Phe124Cys 5-HT_1B_-R variant (Cys/Phe genotype) modifies 5-HT-induced constriction (mediated not only by 5-HT_1B_ but also by co-expressed 5-HT_2A_-Rs). It was shown that in arteries from Cys/Phe individuals, the contribution of 5-HT_1B_-Rs to the mediation of the effects of 5-HT was increased (Verheggen et al. 2006; Table 1).

5-HT_2C_-Rs are widely expressed in the central nervous system and appear to play an important role in psychiatric disorders and drug dependence (Giorgetti and Tecott 2004; Chagraoui et al. 2016). The pre-mRNA of the 5-HT_2C_-R undergoes post-transcriptional editing resulting in diversity among RNA transcripts and 5-HT_2C_-Rs are heterogeneous due to alternative splicing (Werry et al. 2008; Bass 2002; Wang et al. 2000). In addition, a single-nucleotide polymorphism (SNP) in the 5-HT_2C_-R gene, leading to substitution of cysteine 23 to serine (Cys23Ser) in the N-terminal domain of the 5-HT_2C_-R (Table 1, Fig. 5), had been found to be associated with neuropsychological diseases (Lerer et al. 2001) and to alter the response to clozapine (Segman et al. 1997). An allele frequency of about 13% has been found for this variant. Since published results concerning the functional properties of the two isoforms were inconsistent, Manfred Göthert examined, in more detail, the wild-type and the Cys23Ser variant of the 5-HT_2C_-R in transiently transfected HEK293 cells with respect to function (by an aequorin luminescence-based Ca^2+^ assay) and to surface expression (by means of ^3^H-mesulergine binding) (Walstab et al. 2011). Surface expression of the Cys23Ser variant was found to be 116% of that of the wild-type receptor. No difference was observed between wild-type and variant receptor concerning 5-HT-induced increase in cytosolic Ca^2+^ and its inhibition by the inverse agonist SB206553. Furthermore, no difference between wild-type and variant receptor was observed in the time-dependent reduction of 5-HT-induced increase in cytosolic Ca^2+^, i.e. of the rapid and strong receptor desensitization due to preexposure of the cells to 5-HT. On the other hand, prolonged preexposure to SB206553 caused resensitization of the receptor, i.e., elevation of the Ca^2+^ response. However, at the variant receptor, this elevation was seen already within 1 h, whereas at the wild-type receptor, a preexposure time of 4.5 h was needed for this effect to occur. The different time course of SB206553-induced resensitization of the two isoreceptors might be therapeutically relevant for some atypical antipsychotics (such as clozapine) and certain antidepressants (such as mirtazapine) acting as inverse agonists at 5-HT_2C_-Rs. Prolonged preexposure to an inverse agonist is assumed to reduce the constitutive activity of the 5-HT-R in vivo, thereby increasing receptor responsiveness to classical agonists (Walstab et al. 2011).

The human 5-HT_7_-R, first described by Bard et al. (1993), was the most recently identified member of the 5-HT-R family. This receptor, which is coupled to G_s_ protein to stimulate cAMP formation, is expressed in the central nervous system, e.g., in the thalamus, hypothalamus, hippocampus, cerebral cortex, amygdala, and dorsal raphe; it is involved in circadian rhythm by acting at the suprachiasmatic nucleus (Lovenberg et al. 1993) and seems to play a role in the action of antipsychotics and antidepressants (Matthys et al. 2011). Alternative splicing at the second intron, located at the C-terminal end of the 5-HT_7_-Rs, gives rise to three splice variants (5-HT_7(a,b,d)_, Heidmann et al. 1997) which show the same pharmacological properties; among these splice variants, the 5-HT_7(a)_ is the most abundant isoform (Gellynck et al. 2013). All three splice variants have very similar abilities to stimulate adenylyl cyclase in HEK293 cells (Krobert et al. 2001), indicating that the C-terminal tail does not influence ligand binding or G protein coupling. Systematic mutation screening in patients suffering from schizophrenia or bipolar affective disorder revealed an SNP leading to the exchange of proline against leucine in position 279 in the third intracellular loop of the receptor protein (Table 1, Fig. 5; Erdmann et al. 1996); interestingly, both control individuals and patients exhibited the same allele frequency (1%). Manfred Göthert has studied this Pro279Leu variant in comparison to the wild-type receptor in transfected HEK293 cells by means of binding of ^3^H-5-CT to membranes and stimulation of ^3^H-cAMP formation in whole cells evoked by 5-HT-R agonists (Kiel et al. 2003). All agonists and antagonists examined exhibited no difference in affinity between the variant receptor and the wild-type receptor. However, in cells expressing the Pro279Leu variant, the intrinsic activity of all agonists examined in stimulating ^3^H-cAMP accumulation was almost abolished and their potency was 2.9–4.3-fold lower.

The mutation screening study of Erdmann et al. (1996; see above) additionally revealed an SNP leading to the exchange of threonine against lysine in position 92 located in the first transmembrane domain of the receptor protein (Fig. 5). The allele frequency of this variant was <1% for both control individuals and patients (Erdmann et al. 1996). In HEK293 cells transfected either with the wild-type cDNA or that of the Thr92Lys variant, we determined binding of ^3^H-5-CT to membranes and stimulated ^3^H-cAMP accumulation in whole cells (Brüss et al. 2005b). The variant receptor exhibited 3–11 times lower binding affinity to the agonists 5-HT, 5-CT, 8-OH-DPAT, sumatriptan, and RU24969 compared to the wild-type receptor. The variant receptor, however, did not differ from the wild type with respect to the binding properties of antagonists such as risperidone, mesulergine, clozapine, and SB-269970. In agreement with the decreased binding affinity of 5-HT, 5-CT, RU24969, and 8-OH-DPAT for the variant receptor, these agonists were less potent in stimulating ^3^H-cAMP accumulation in cells expressing the variant receptor. Sumatriptan did not stimulate cAMP accumulation in spite of its affinity for both receptor isoforms pointing to a weak antagonistic property of this drug at the 5-HT_7_-R. SB-269970 and clozapine were equipotent at both the variant and the wild-type receptor in producing a rightward shift of the 5-HT concentration-response curve for its stimulant effect on ^3^H-cAMP accumulation. Thus, the results of our two studies (Kiel et al. 2003; Brüss et al. 2005b; Table 1) may have relevance for drugs acting on 5-HT_7_-Rs which affect circadian rhythm.

## Beyond serotonin

### Carbon monoxide toxicology

When Manfred Göthert joined the Institute of Pharmacology at the University of Hamburg in 1967, he first did not deal with serotonin and not even with pharmacology. As a member of the group of G. Malorny, then head of department, the first scientific studies of Manfred Göthert were dedicated to carbon monoxide toxicology. Experiments on *animals* exposed to carbon monoxide revealed that the partial pressure of CO in the pneumoperitoneum (which served to quantify the partial pressure of CO in tissue) is the lower, the higher its affinity for hemoglobin is. This was shown for not only rats (high), guineapigs (high), and rabbits (low hemoglobin affinity) (Göthert et al. 1968, 1970) but also for single rabbits, which for unknown reasons markedly differed in their hemoglobin affinity (Göthert and Malorny 1969). Moreover, the partial pressure of CO in the pneumoperitoneum of rabbits exposed to air containing 1000 parts per million (ppm) of CO at normal (1 bar) or increased pressure (3 or 4 bar) was higher in the former than in the latter groups (Gerhardt et al. 1972). Although these findings may appear strange at first glance, they can easily be explained by the law of mass action; carbon monoxide and oxygen compete for the same receptor (hemoglobin).

CO toxicology was also studied in *humans*. In volunteers who breathed air containing 50 ppm CO under normal (1 bar) and hyperbaric (3 bar) pressure over a time period of 2 h, the percentage of CO hemoglobin in the group with normal pressure (7%) was higher than in the group with increased pressure (5%) (Gerhardt et al. 1971). This result is practically relevant since the possibility had to be considered that Caisson workers might suffer from an increased CO-hemoglobin concentration. On the basis of this study, a reduction of the threshold limit value (maximale Arbeitsplatzkonzentration, MAK) for CO in Caisson workers did not appear to be necessary. In another study (Bender et al. 1971), human volunteers exposed to air containing 100 ppm CO over a time period of 2.5 h showed a CO concentration of 55 ppm in the alveolar air and exhibited reduced visual perception, manual dexterity, and ability to learn and to perform certain intellectual tasks. This study clearly confirmed that the reduction of the MAK for CO in Germany from 100 to 50 ppm in 1966 was really justified.

### Ethanol, general anesthetics, gabapentinoids, and ion channels

#### General anesthetics, alcohols, and ligand-gated cation channels

In parallel to the toxicological studies on CO, Manfred Göthert became interested in the mode of action of general anesthetics. Although this work is apparently unrelated to 5-HT-Rs, it was an important period of his work and Manfred Göthert remained life-long interested in the cardiovascular and neuronal effects of general anesthetics and alcohols. Specifically, this early work was continued in Bonn 15 years later with K. Fink on ionotropic glutamate-Rs and with H. Bönisch, M. Brüss, and M. Barann on 5-HT_3_-Rs. In the early 70s, not much was known on the mode of action of general anesthetics; while it is even today not completely clear for inhalational anesthetics, for the injectable anesthetics propofol and etomidate, the GABA_A_-R with a specific subunit composition has been found as the predominant target. According to the current understanding, all ligand-gated ion channels are targets of anesthetic drugs, however, in a more specific way than general hydrophobic interaction with cell membrane or membrane proteins according to the Meyer-Overton correlation (for review, see Franks 2006; Lynch 2008). Most attention for all anesthetics has been attracted by not only interactions with the GABA_A_-R but also the other ligand-gated ion channels as well as Na^+^ and K^+^ channels (Rudolph and Antkowiak 2004; Hemmings et al. 2019). After diethylether had been widely administered as an anesthetic since Morton’s demonstration in the Massachusetts General Hospital in Boston in 1846, halothane was introduced in Germany in 1956, methoxyflurane in 1960, and enflurane in 1966. These inhalative anesthetics were frequently used in anesthesiology around 1970 when Manfred Göthert started to work on it; obviously other inhalative anesthetics such as isoflurane, desflurane, or sevoflurane that were less metabolized followed later.

Manfred Göthert presented first results of this work at the 10th Spring Meeting of the German Pharmacological Society in Mainz 1969 (Göthert and Benthe 1969). In diethylether-anesthetized guinea-pigs or rats, noradrenaline and adrenaline concentrations increased in the heart but decreased in the adrenal medulla, whereas in halothane anesthesia, noradrenaline only initially decreased in the heart and in chloroform anesthesia, noradrenaline decreased in the heart for the entire duration of application (Göthert 1971). It explained the overall less pronounced cardiosuppressive effects of diethylether vs. chloroform or halothane but did not reveal the underlying mode of action of these general anesthetics. In a series of further studies, he provided an in-depth analysis of the differential effects of diethylether, chloroform, halothane, enflurane, or methoxyflurane on catecholamine release in the adrenal medulla and myocardium and the resulting cardiovascular effects (Göthert and Tuchinda 1973; Benthe et al. 1973; Göthert et al. 1974a, b; Schmoldt and Göthert 1974). The overall negative chronotropic effect of halothane could be attributed to the inhibition of catecholamine release from adrenal medulla (30%), the inhibition of noradrenaline release from sympathetic nerve terminals in the heart (25%; Table 3), a direct effect on the myocardium (25%), and a stimulatory effect on parasympathetic nerves (20%; Göthert and Tuchinda 1973). In vitro in cat heart, the order of negative ionotropic effects, i.e., halothane > chloroform > diethylether, was confirmed (Benthe et al. 1973). In 1974, he showed the inhibition of ACh-stimulated catecholamine release from isolated bovine adrenal medulla by inhalation anesthetics (halothane (Table 3), methoxyflurane, chloroform) and aliphatic alcohols (n-propanol, n-butanol, n-pentanol, n-hexanol) and concluded, as mode of action, a hydrophobic interaction with membranes correlating to the membrane-buffer partition coefficients (Göthert et al. 1974a). The catecholamine release from cat adrenal medulla after splanchnic nerve stimulation was largely reduced by chloroform and ether (Göthert et al. 1975). However, this could not be attributed at in vivo applied anesthetic concentrations to a reduced catecholamine synthesis in the adrenal medulla (Schmoldt and Göthert 1974). The spontaneous release of catecholamines from the adrenal medulla was concentration-dependently decreased by halothane (Göthert and Dreyer 1973) and, similarly, by methoxyflurane (Dreyer et al. 1974). Halothane decreased the coronary flow in the isolated rabbit heart perfused at constant pressure (Göthert and Guth 1975) presumably by reducing the vasoconstrictor effect of noradrenaline on coronary arteries. As only the nACh-R-mediated noradrenaline release from myocardial sympathetic nerve terminals was inhibited by halothane and not the release induced by high K^+^, he concluded that halothane might cause a conformational change most potently of the nACh-R protein (Göthert et al. 1974b; Göthert 1974; Table 3), an idea which is still assumed to be a major part of the anesthetics’ mechanism. In the adrenal medulla, he located the effect of halothane to the cell membrane of chromaffin cells or, more specifically, to membrane proteins such as the nACh-R or, at lower potency, GABA-Rs and 5-HT-Rs (Göthert et al. 1976a; Table 3). An investigation of the mode of action of the cardiovascular effects of enflurane resulted in the same conclusion, i.e. that enflurane interacted with hydrophobic regions of the nACh-R while mACh-Rs were much less sensitive to enflurane (Göthert and Wendt 1977a, b).

**Table 3:** Effect of halothane, pentobarbital, and ethanol on the evoked catecholamine release from peripheral organs and brain tissue

		Ligand-gated cation channels				Activation of G_q_ protein-coupled receptors		Voltage-dependent cation channels			
		NMDA	Kainate	nACh	5-HT_3_	Histamine H_1_	mACh	K^+^	Veratridine	Electrical stimulation	
		IC_50_									
Halothane	^1^Bovine adrenal medulla			0.25	4.6	> 4.3	> 4.3	> 14			mM
Halothane	^2^Rabbit heart			0.06				> 1		> 1	mM
Pentobarbital	^3^Rabbit heart			34				190		440	μM
Ethanol	^4^Rabbit heart			129	203			830		1150	mM
Ethanol	^5^Human cortex	90	115					> 150	> 150		mM
Ethanol	^6^Rat cortex	45	126					> 320		> 320	mM

To evoke catecholamine release from bovine adrenal medulla chromaffine cells and noradrenaline release from noradrenergic nerve terminals, intracellular Ca^2+^ was increased by activation of ligand-gated or voltage-dependent cation channels or of G_q_ protein-coupled receptors. The table shows that the inhibitory potency of the three agents (IC_50_, concentration leading to an inhibition of 50%) at ligand-gated channels is higher than at G_q_ protein-coupled receptors and voltage-dependent cation channels. Within the ligand-gated ion channels, the potency towards NMDA receptors is higher than that towards nACh and 5-HT_3_ receptors. Inhibition of the nACh receptor-mediated inhibition of catecholamine/noradrenaline release may contribute to the clinical effect of halothane and pentobarbital. Thus, the IC_50_ of *halothane* is close to its MAC in saline of 0.24 mM (the MAC (minimal alveolar concentration) is a parameter of the potency of a general anesthetic). The IC_50_ of *pentobarbital* is within the range of the plasma/serum concentration (4.4-44 μM) obtained under treatment with this barbiturate. Inhibition of the NMDA-induced noradrenaline release in human cortex may contribute to the toxic effect of *ethanol* 2‰ (= 46 mM; inhibition by ~ 40% at this concentration). Derived from ^1^Göthert (1972) and Göthert et al. (1976a), ^2^Göthert (1974), ^3^Göthert and Rieckesmann (1978), ^4^Göthert and Thielecke (1976) and Göthert et al. (1979a), ^5^Fink et al. (1992a), and ^6^Göthert and Fink et al. (1989) and Fink and Göthert (1990)

Besides the work on inhalative anesthetics, Manfred Göthert also studied the mode of action of the injectable anesthetic pentobarbital where he also identified as the most potent effect the inhibition of nACh-R-mediated noradrenaline release from rabbit cardiac sympathetic nerves which is still in line with current understanding of barbiturate action (Ye and Ewing 2018); the inhibition of high K^+^ or electrically evoked noradrenaline release occurred at 10-30 times higher pentobarbital concentrations (Göthert and Rieckesmann 1978; Table 3).

**Fig. 6:**
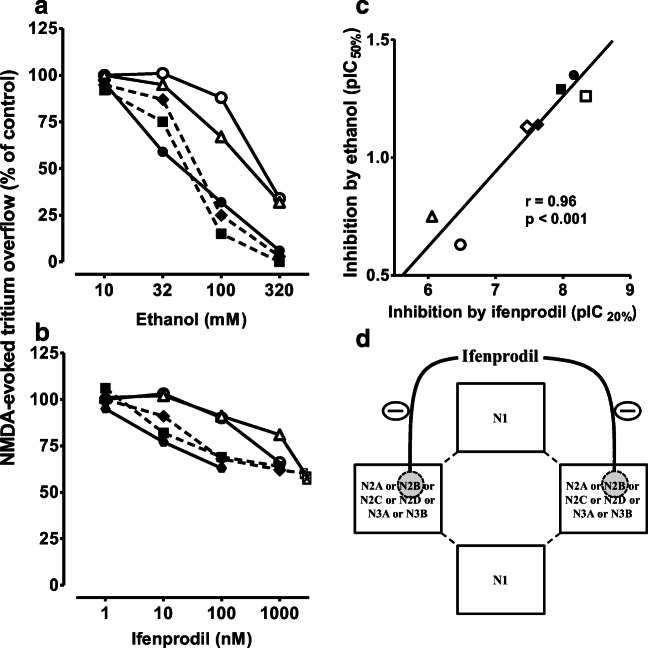
Effect of ethanol and ifenprodil on the NMDA-evoked release of various transmitters from rat cerebral cortex and/or striatal slices. **a** and **b** The concentration-response curves (CRCs, SEM values not shown). The negative logarithms of the concentrations causing the half-maximum effect, i.e. an inhibition by 50% (ethanol, pIC_50%_) and by 20% (ifenprodil, pIC_20%_), were correlated with each other as shown in **c**, yielding a correlation coefficient close to 1. This suggests that ethanol acts on NMDA receptors containing an N2B subunit which is inhibited by ifenprodil in noradrenergic, serotoninergic, and GABAergic neurones. *Closed circles*, cortical slices preincubated with ^3^H-noradrenaline; *closed squares*, cortical slices, ^3^H-5-HT; *closed rhomboids*, cortical slices, ^3^H-GABA; *open circles*, striatal slices, ^3^H-dopamine; *open triangles*, striatal slices, ^3^H-choline; *open squares*, striatal slices, ^3^H-5-HT (CRCs not shown); *open rhomboids*, striatal slices ^3^H-GABA (CRCs not shown). Re-drawn from Fink and Göthert (1996). **d** The structure of NMDA receptors (which occur as di-heteromeric or tri-heteromeric tetramers) and the site of action of ifenprodil. *GABA*, γ-aminobutyric acid; *NMDA*, N-methyl-D-aspartate

In 1976, he extended the idea of a hydrophobic interaction of the inhalative anesthetics with the nACh-R to the alcohols ethanol, 1-propanol, 1-butanol, and 1-pentanol using dimethylphenylpiperazine-induced or ACh-induced noradrenaline release from isolated perfused rabbit heart; he suspected an altered agonist-receptor interaction (Göthert and Kennerknecht 1975; Göthert et al. 1976b). In these experiments, he found a threshold concentration of 36 mM ethanol for the inhibitory effect on nACh-Rs which may be achieved in in vivo intoxications (Göthert and Thielecke 1976; Table 3). After Manfred Göthert had become Professor of Pharmacology in Bonn, this work was continued on another ligand-gated ion channel, the N-methyl-D-aspartate (NMDA)-R. K. Fink and Manfred Göthert established and characterized the model of NMDA-R-mediated noradrenaline release in brain slices (Fink et al. 1989, 1990c) and supported it by radioligand binding studies (Fink et al. 1992c). They used the functional approach of NMDA-evoked noradrenaline release to demonstrate the inhibitory effect of ethanol on NMDA-Rs. The threshold concentration was between 10 and 32 mM which was definitely in the range of clinical ethanol intoxications (Göthert and Fink 1989; Table 3, Fig. 6). The experiments were repeated with the aliphatic alcohols methanol, ethanol, 1-propanol, 1-butanol, 1-pentanol, and 1-hexanol and demonstrated a strong correlation of the alcohols’ membrane-buffer partition coefficients and their inhibitory potencies (Fink and Göthert 1990, 1991); ethanol turned out slightly more potent than the overall correlation between hydrophobicity and potency indicating a more specific interaction. As soon as they identified presynaptic NMDA-Rs on noradrenergic axonal terminals in rat neocortex using a synaptosomal preparation (Fink et al. 1990b; Göthert and Fink 1991), they could also demonstrate the inhibitory effect of ethanol on presynaptic NMDA-Rs (Fink and Göthert 1992). The existence of the ionotropic glutamate-Rs NMDA, kainate, and AMPA (α-amino-3-hydroxy-5-methyl-4-isoxazolepropionic acid) was also shown in the *human* neocortex, including the inhibitory effects of ethanol at concentrations that were observed in human intoxications in vivo (Fink et al. 1992a, b; Table 3). All these data of Manfred Göthert’s group contributed strong evidence to the idea that general anesthetics and alcohols have rather individual effects on different neuronal target structures as compiled in box 2 of the review by Rudolph and Antkowiak (2004).

Although the interaction may be partially or even predominantly hydrophobic by nature, it still depends on size, type, and exposition of the hydrophobic patches or uncharged sequences of the individual receptor protein. In order to explain the differing sensitivities to ethanol of NMDA-Rs on noradrenergic, serotonergic, GABAergic, dopaminergic, and cholinergic neurones, K. Fink and Manfred Göthert compared the ethanol effects to the respective effects of the NR2B (current nomenclature GluN2B) subunit preferential receptor antagonist ifenprodil. NMDA-Rs are tetramerically composed of two obligatory GluN1 and two GluN2 or GluN3 subunits; GluN2 exists in four subtypes A-D and GluN3 in two subtypes A and B (Paoletti et al. 2013) while the specific role of different NMDA-R assemblies is not completely clear. They found a strong correlation (0.96; *p*<0.001) of ethanol and ifenprodil potency and concluded that ethanol predominantly inhibits NR2B-assembled NMDA-Rs on noradrenergic, serotoninergic, and GABAergic neurones (Fink and Göthert 1996) (Fig. 6).

During these experiments, the potential effect of hyperosmolarity was discussed within the group and, as few agents e.g. sugars, urea, and ethanol can cause serious hyperosmolar disorders in patients, the question was addressed with D-glucose. Interestingly, high K^+^-evoked GABA release in neocortex was strongly increased by D-glucose ≥32 mM, that of acetylcholine was unaffected, and that of noradrenaline and 5-HT was decreased (Fink and Göthert 1993a). The increased GABA release remained elusive but might result from the blockade of ATP-sensitive K^+^ channels by increased ATP levels. It was concluded that an interaction between GABAergic interneurones and other neurones downstream was the underlying mechanism that increased GABA release and that this phenomenon could explain some of the symptoms in hyperosmolar diabetic coma (Fink et al. 1994a). The group also proved the modulation of NMDA-R-mediated noradrenaline release by presynaptic α_2_-ARs and H_3_-Rs (Fink and Göthert 1993b; Fink et al. 1994b; Table 4) as well as the modulation of NMDA R-mediated 5-HT release (Fig. 3) by 5-HT autoreceptors (Fink et al. 1996) and presynaptic α_2_-heteroreceptors (Fink et al. 1995b).

**Table 4:** Presynaptic receptors on central and peripheral noradrenergic neurones identified by Manfred Göthert. For presynaptic 5-HT receptors, see text and Tables 1 and 2

Effect	Modulator (receptor)		Tissue	References
Inhibition	Noradrenaline (α_2_)	*Basic evidence*	Human saphenous vein, pulmonary artery, corpus cavernosum; rabbit heart, aorta, pulmonary artery; rat cerebral cortex	Göth ert et al. (1984), Hentrich et al. (1986), Molderings et al. (1989b); Göthert (1977), Docherty et al. (1982); Göthert et al. (1979b), Fink and Göthert (1993b)
		*α*_*2A*_*-subtype*	Human right atrium, saphenous vein; rabbit pulmonary artery	Molderings and Göthert (1995a), Molderings et al. (2000a, 2003a), Brüss et al. (2003)
	Imidazoline (LPA_1_ and/or LPA_3_?)		Human atrium, pulmonary artery; rabbit aorta, pulmonary artery; guinea-pig pulmonary artery; rat aorta and vena cava; *rat pheochromocytoma cell line PC12*	Likungu et al. (1996), Molderings et al. (1997); Docherty et al. (1982), Göthert and Molderings (1991), Molderings et al. (1991), Molderings and Göthert (1995b); Molderings and Göthert (1999); Molderings and Göthert (1998); Molderings et al. (2002a)
	Acetylcholine (mACh)		Rabbit heart, pulmonary artery	Göthert (1977), Molderings et al. (2006)
	Histamine (H_3_)		Human cerebral cortex, saphenous vein; pig retina vasculature; rat cerebral cortex; mouse cerebral cortex	Schlicker et al. (1994c), Molderings et al. (1992a); Schlicker et al. (1990); Schlicker et al. (1989c, 1992b, 1994c), Fink et al. (1994b); Schlicker et al. (1992b, c, 1994b, c), Kathmann et al. (1994), Nickel et al. (2001)
	Opioid μ and δ		Rat cerebral cortex	Göthert and Wehking (1980), Göthert et al. (1979b)
	Somatostatin		Rat hypothalamus	Göthert (1980b)
	Prostaglandin E_2_ (EP_3_)		Human right atrium, pulmonary artery, saphenous vein, corpus cavernosum	Molderings et al. (1992b, 1994a, 1998a)
	Cannabinoid (CB_1_)		Human, guinea-pig hippocampus; guinea-pig vessels	Schlicker et al. (1997b), Kathmann et al. (1999); Schultheiß et al. (2005)
Facilitation	Noradrenaline, adrenaline (β_2_)	*Basic evidence*	Human pulmonary artery, saphenous vein; rat vena cava	Göthert and Hentrich(1985), Hentrich et al. (1985), Molderings et al. (1988b); Göthert and Kollecker (1986)
		*via AT*_*1*_ *receptors*	Human saphenous vein; rat vena cava, *pithed rat*	Molderings et al. (1988b); Göthert and Kollecker (1986), Schlicker et al. (1988c)
	Acetylcholine (nACh)		Rabbit heart	Göthert (1974)
	Glutamate	(NMDA)	Human, guinea-pig, rat cerebral cortex	Fink et al. (1992b), Kathmann et al. (1999), Fink et al. (1990b)
		(AMPA)	Human, rat cerebral cortex	Fink et al. (2000, 2002b)
	Angiotensin II (AT_1_)		Human pulmonary artery, saphenous vein; rabbit heart	Molderings et al. (1988b); Göthert (1977)
	ACTH (MC_2_)		Rabbit pulmonary artery	Göthert (1981, 1984), Göthert and Hentrich (1984)
	Prostaglandin D_2_ (DP)		Human right atrium, pulmonary artery, saphenous vein, corpus cavernosum	Molderings et al. (1992b, 1994a, 1998a)

#### Gabapentinoids and voltage-gated Ca^2+^ channels

**Fig. 7:**
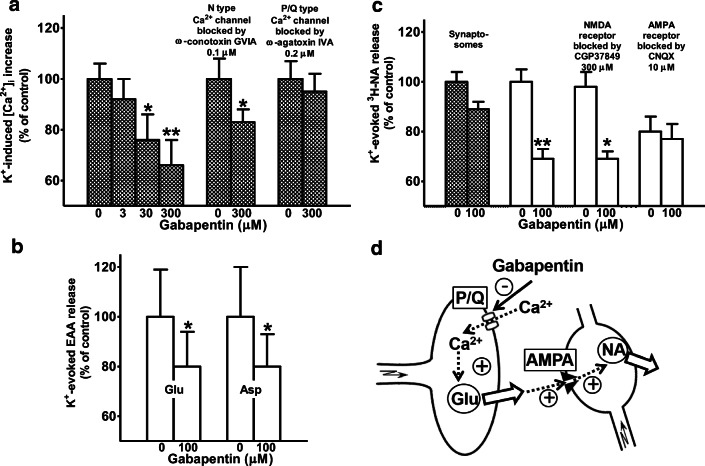
Chain of events involved in the inhibitory effect of gabapentin on noradrenaline (NA) release in rat brain cortex. Gabapentin inhibits the K^+^-induced **a** Ca^2+^ influx via P/Q-type (but not N-type) Ca^2+^ channels, **b** glutamate and aspartate release, and **c** NA release via AMPA (but not NMDA) receptors. Experiments were performed on slices or synaptosomes (dotted columns) and results are expressed as means ± SEM (*p < 0.05, **p < 0.01, based on the *t*-test for paired (B) or unpaired (A, C) data). The fact that the inhibitory effect of gabapentin on NA release (C) was not retained in isolated nerve endings (synaptosomes) demonstrates that it is not related to a direct effect on the noradrenergic neurone. The effect of gabapentin occurred in the range of therapeutically relevant plasma concentrations of 10–100 μM. Re-drawn from Fink et al. (2000). The experiments were further elaborated in the study by Fink et al. (2002b), which shows that the mechanisms also occur in human cortical slices and also extend to pregabalin, another gabapentinoid, but not to its enantiomer R-(-)-3-isobutylgaba. The schematic drawing in **d** shows that gabapentin (and pregabalin) (i) inhibit Ca^2+^ influx into glutamatergic neurones via P/Q-type (Ca_V_2.1) voltage-gated Ca^2+^ channels by binding to its α_2_δ subunit, subsequently leading to (ii) decreased glutamate release, (iii) reduced activation of excitatory AMPA receptors on noradrenergic neurones, and (iv) eventually to a decrease in NA release. *AMPA*, α-amino-3-hydroxy-5-methyl-4-isoxazolepropionic acid; *CNQX*, 6-cyano-7-nitroquinoxaline-2,3-dione; *EAA*, excitatory amino acids; *NMDA*, N-methyl-D-aspartate

A further chapter of work focussed on presynaptic voltage-gated Ca^2+^ channels. Using Ca^2+^ fluorometry on synaptosomes, the PhD student W. Meder identified the P/Q-type (meanwhile referred to as Ca_V_2.1 α_1_ subunit) voltage-gated Ca^2+^ channel as the major and the N-type (Ca_V_2.2 α_1_ subunit) and the Na^+^/Ca^2+^ exchanger as minor contributors to presynaptic Ca^2+^ entry in rat and human neocortex (Meder et al. 1997, 1999; Fink et al. 2002a). In the same paradigm, the mode of action of the gabapentinoids gabapentin and pregabalin was discovered, which inhibit P/Q-type (Ca_V_2.1) voltage-gated Ca^2+^ channels by binding to its α_2_δ subunit resulting downstream in attenuated glutamate/aspartate release and, thus, less activation of AMPA-R input on noradrenergic terminals (Fink et al. 2000, 2002b) (Fig. 7). The latter work has been cited by 549 articles (Google Scholar, accessed on March 26, 2021), 3 patents since it appeared (ResearchGate.net). It was again the result of a close collaboration with, in this case, Parke-Davis and, after its acquisition, with Pfizer; the work was initially triggered by Feuerstein’s finding that ω-conotoxin GVIA inhibited noradrenaline and acetylcholine release in the human neocortex (Feuerstein et al. 1990), an effect, which could now be explained.

### Various presynaptic receptors, imidazolines, and agmatine

#### Various presynaptic receptors

Noradrenaline attracted the attention of Manfred Göthert even earlier than 5-HT did. In a series of studies, structure-activity relationships were determined for compounds provided by the Beiersdorf company (Hamburg) with respect to their noradrenaline-depleting effect (Benkert et al. 1975) or their antagonistic effects at α_1_-ARs and/or α_2_-ARs (Benthe et al. 1972; Göthert et al. 1983b; Schlicker et al. 1984b). The α_1_-AR antagonist BE 2254 played some role as drug tool in own studies (e.g., Göthert et al. 1981b) or studies from other groups (e.g., ^125^I-BE 2254 in Engel and Hoyer 1981). However, Manfred Göthert became particularly interested in presynaptic autoreceptors and heteroreceptors on noradrenergic neurones. In the same year, in which he entered this field (Göthert 1977), long reviews about numerous types and sites of presynaptic receptors written by key players appeared (Langer 1977; Starke 1977; Westfall 1977); nonetheless, he became one of the major scientists in this area of research.

In the *central nervous system*, α_2_-autoreceptors, e.g. in the rat (Göthert et al. 1979b) and mouse cerebral cortex (Schlicker et al. 1992b), and particularly heteroreceptors attracted his attention (Table 4); he was supported in this respect by E. Schlicker and K. Fink and later by M. Kathmann. In detail, he dealt with histamine, opioid, somatostatin, and cannabinoid receptors (the latter ones will be described in the “Cannabinoids” section; Table 4). Presynaptic histamine H_3_-Rs, originally identified as autoreceptors by Arrang et al. (1983), were identified for the first time on serotoninergic, noradrenergic, and dopaminergic neurones of the brain (Schlicker et al. 1988b, 1989c, 1993; Fink et al. 1990a; Fig. 3, Table 4). Although most studies were carried out on rodent brain, the H_3_-R was also identified on the noradrenergic neurones of the human cerebral cortex (Schlicker et al. 1994c). Its counterpart in the mouse brain cortex was examined in detail. (i) In a mechanistic study, the receptor-mediated inhibitory effect was the more pronounced the lower the Ca^2+^ concentration in the medium or the stimulation frequency was; moreover, experiments with N-ethylmaleimide (Schlicker et al. 1994b) and pertussis toxin (Schlicker et al. 1994c) suggested that this receptor is G_i/o_ protein-coupled. The latter findings were interesting since the H_3_-R was cloned 5 years later only (Lovenberg et al. 1999). (ii) A receptor interaction, like that shown for the r5-HT_1B_-R in the rat vena cava (Molderings and Göthert 1990; see the “Presynaptic serotonin heteroreceptors on noradrenergic neurones” section), was also shown for the H_3_-R in the mouse brain. Pre-activation of the α_2_-autoreceptor decreases the inhibitory effect mediated via the H_3_-R; the reverse is true as well (Schlicker et al. 1992b). Since α_2_-autoreceptor blockade consequently increases the extent of the H_3_-R-mediated effect, subsequent experiments were usually performed in the presence of the α_2_-AR antagonist rauwolscine. (iii) The potencies of new H_3_-R antagonists/inverse agonists were compared to their affinities in radioligand studies in the mouse brain (Nickel et al. 2001). The compounds had been synthesized by W. Schunack and H. Stark (Berlin), who later developed the H_3_-R inverse agonist pitolisant together with J.C. Schwartz (Paris), which has been marketed as a novel drug against narcolepsy in 2016 (Ganellin et al. 2018). A typical property of H_3_-Rs is their constitutive activity (Rouleau et al. 2002) and accordingly the H_3_-R inverse agonist pitolisant tended to increase noradrenaline in mouse brain cortex slices (reviewed in Schlicker and Kathmann 2016). The possibility has to be considered that this effect (provided that it also occurs in human brain) contributes to the main action and/or the side effects of pitolisant.

Somatostatin not only serves as an inhibitor of the release of a series of hormones including growth hormone but also occurs in several brain regions (Günther et al. 2018). In a paper that appeared in *Nature*, Göthert (1980b) was the first to show that somatostatin also acts via presynaptic inhibitory receptors. This effect, which was examined in rat brain slices, is selective in two respects. First, somatostatin inhibits noradrenaline release in the hypothalamus but not in the cerebral cortex. Second, the inhibitory effect of somatostatin does not extend to 5-HT release.

*Peripheral* presynaptic receptors on postganglionic sympathetic neurones attracted the attention of Manfred Göthert already in Hamburg and later in Essen (supported by F. Hentrich) and Bonn (supported by G. Molderings). Unlike in the brain, the peripheral noradrenergic neurones are equipped with a series of facilitatory receptors. Six of them have been considered, including two ligand-gated ion channels (5-HT_3_, Tables 2 and 3 and nACh-R, Tables 3 and 4), one G_q_-coupled-receptor (AT_1_; Table 4) and three G_s_-coupled receptors (β_2_, MC_2_, and DP; Table 4). For each of the four G protein-coupled receptors, at least one human model has been described.

The presynaptic β_2_-AR was examined with respect to its location, mechanism, and physiological role. (i) In two in vitro models and in one in situ model (Table 4), the effect of a β-AR agonist was counteracted by inhibitors of the angiotensin-converting enzyme and/or angiotensin AT_1_-R antagonists. These data suggest that at least part of the β_2_-ARs is not located directly on the postganglionic sympathetic nerve endings but rather in the wall of blood vessels. When activated by a β_2_-AR agonist, the receptors lead to an increased formation of angiotensin II, which in turn facilitates noradrenaline release via presynaptic AT_1_-R. In the rat vena cava and human saphenous vein, the occurrence of AT_1_-Rs has been shown directly (Göthert and Kollecker 1986; Molderings et al. 1988b). (ii) Hentrich et al. (1985) showed in the human pulmonary artery that a stimulator of cAMP formation, an inhibitor of its degradation, and a lipid-soluble cAMP analog increase noradrenaline release. The facilitatory effect of the β-AR agonist isoprenaline on noradrenaline release was markedly enhanced in the presence of a low concentration of the stimulator of cAMP formation, suggesting that the β_2_-AR is coupled to adenylate cyclase. (iii) The possibility that the β_2_-AR, reached by endogenous adrenaline, is involved in a positive feedback loop and may even be implicated in the development of essential hypertension had to be considered (for review, see Rand and Majewski 1984). If this were true also for the human saphenous vein, a β-AR antagonist should decrease noradrenaline release. Such an effect, however, did not occur, even if the veins were preincubated with ^3^H-adrenaline instead of ^3^H-noradrenaline (Molderings et al. 1988a).

Göthert (1981) was the first to identify a presynaptic ACTH-R. In experiments similar to those described in the previous paragraph, evidence was presented that also the presynaptic ACTH-R, which was identified on the postganglionic neurones of the rabbit pulmonary artery, is positively coupled to adenylate cyclase (Göthert and Hentrich 1984). Although evidence for a facilitatory receptor for prostaglandin D_2_ (DP-R) was first presented by Nakajima and Toda (1984) in canine mesenteric arteries, final proof for their existence based on an appropriate antagonist was given for four human tissues in the lab of Manfred Göthert (Table 4). Interesting enough, in each of the four tissues, both facilitatory (DP) and inhibitory (EP_3_) prostaglandin-Rs could be identified (Table 4).

In addition to the EP_3_-R, other types of inhibitory heteroreceptors and the inhibitory α_2_-autoreceptor have been identified on peripheral noradrenergic neurones (most receptor types also in human tissues; Table 4). For the α_2_-autoreceptor in the human saphenous vein and the rabbit pulmonary artery, the potencies of α_2_-AR antagonists in functional experiments were correlated with their affinities in radioligand binding studies on tissues or cells expressing one α_2_-subtype only (Molderings and Göthert 1995a). The study clearly showed that the autoreceptor is α_2A_ both in the human and in the rabbit vessel. These results conform to two general observations, namely (i) that presynaptic α_2_-ARs belong to the α_2A_-subtype in species like humans and rabbits, whereas in guinea pigs and rodents, the α_2D_-subtype (which turned out to be the species homolog of α_2A_) is involved instead (Trendelenburg et al. 1997) and (ii) that, compared to the α_2A/D_-subtype, the α_2B_-subtype, and/or α_2C_-subtype, plays no or a smaller role only (Brede et al. 2004). Nonetheless, the α_2A_-autoreceptors in humans and rabbits do not possess completely identical properties since rilmenidine and oxymetazoline behave as antagonists in humans but as agonists in rabbits (Molderings et al. 2000a, 2003a). This is also reflected by marked differences in the amino acid sequence of the two species homologs (Molderings et al. 2000a; Brüss et al. 2003).

#### Imidazolines

For the identification of α_2_-ARs, ligands with imidazoline structure like the agonist clonidine and the antagonist BDF 6143 have been used frequently. In addition to their effects on α_2_-ARs, imidazolines appear to possess α_2_-AR-independent effects and in this context, (i) imidazoline recognition sites (Göthert et al. 1983a), (ii) presynaptic imidazoline receptors, and (iii) imidazoline I_1_ and I_2_ binding sites attracted the attention of Manfred Göthert. The latter two topics, which were elaborated together with G.J. Molderings and reviewed repeatedly (Göthert et al. 1995b, 1999; Molderings and Göthert 1999; Molderings et al. 1995b, 1999a), will be considered here in more detail.

The study of Docherty et al. (1982), a cooperation project between Manfred Göthert and K. Starke, was an early hint to the occurrence of *presynaptic imidazoline-Rs* in addition to α_2_-ARs in rabbit aorta and pulmonary artery. BDF 6143, an α_2_-AR antagonist with imidazoline structure, concentration-dependently facilitated, did not affect or even inhibited noradrenaline release, whereas a pure inhibitory effect occurred when the α_2_-ARs had been blocked by rauwolscine, an α_2_-AR antagonist devoid of imidazoline structure. Göthert and Molderings et al. (1991) showed that imidazolines like the α_1_-AR agonist cirazoline, the α_2_-AR agonist clonidine, and the α_2_-AR antagonists idazoxan and phentolamine inhibited noradrenaline release in the presence of rauwolscine. The latter proved to be antagonistic not only against α_2_-ARs (high pA_2_ > 8) but also against imidazoline-Rs (low pA_2_ < 7; Molderings et al. 1991). Further studies revealed that the potencies of the imidazoline-R agonists are not correlated with their lipophilicity (log P) and their affinities at the imidazoline I_1_ and I_2_ binding sites discussed below (Molderings and Göthert 1995b). Presynaptic imidazoline-Rs, although not found in the rabbit or rat brain (Schlicker et al. 1997c), were also identified in human, guinea-pig, and rat cardiovascular tissues (Table 4). In addition to high concentrations of rauwolscine, also high concentrations of the cannabinoid CB_1_-R antagonists rimonabant (former name SR141716) and LY320135 showed an antagonistic effect at the presynaptic imidazoline-Rs (Molderings et al. 1999b).

Presynaptic imidazoline-Rs were also found in the rabbit heart (Fuder and Schwarz 1993), the rat kidney (Bohmann et al. 1994), and, using an electrophysiological technique, in rat superior cervical ganglion neurones (Chung et al. 2010). Nonetheless, the evidence is not unequivocal; Gaiser et al. (1999) re-investigated the effects of 10 α-AR and/or imidazoline-R agonists in the rabbit pulmonary artery using conditions under which an endogenous α_2_-AR-mediated auto-inhibition of noradrenaline release does not occur. In their study, rauwolscine revealed the same potency against each agonist (pA_2_ ~ 8) leaving no place for presynaptic imidazoline-Rs. However, Molderings et al. (2002a, b) found another example of a functional imidazoline-R in a cell line, i.e. pheochromocytoma 12 cells (PC12) of rats, which possess many properties of sympathetic neurones but are devoid of α_2_-AR and CB_1_-R mRNA. The inhibitory effect of imidazolines was shared by 1-oleoyl-lysophosphatidic acid which activates LPA_1_-Rs and LPA_3_-Rs (former designation edg2 and edg7, respectively; Chun et al. 2010). mRNA of the latter two receptors, which are the only G protein-coupled receptors with a significant homology (of ~ 40%) to α_2_-Rs and CB_1_-Rs, could indeed be detected in PC12 cells (Molderings et al. 2002a). LPA_1_-Rs and/or LPA_3_-Rs may represent the molecular entities of the presynaptic imidazoline-Rs; unfortunately, final proof based on knockout mice is so far missing.

*Imidazoline binding sites*, which are pharmacologically different from α-ARs and presynaptic imidazoline-Rs, were identified first in bovine ventrolateral medulla (I_1_) and human fat cells (I_2_); a third type of imidazoline binding site (I_3_) was suggested as well (reviewed in Regunathan and Reis 1996). In collaboration with G.J. Molderings, Manfred Göthert further characterized imidazoline sites in a threefold manner. (i) The occurrence of imidazoline sites in various preparations was shown by radioligand binding studies. Using ^3^H-clonidine and ^3^H-idazoxan, both I_1_ (Molderings et al. 1993) and I_2_ sites (Molderings et al. 1994b) were found in bovine adrenal medulla, i.e. in a tissue which is devoid of α_2_-ARs. Using the same radioligands, I_1_ sites were also identified in the aforementioned PC12 cells (Molderings et al. 2007a) and I_2_ sites in rat and human stomach (Molderings et al. 1998b). Experiments dedicated to further disclose the properties of the I_1_ sites in PC12 cells showed that the ligands under study had a similar affinity for ^3^H-clonidine and ^3^H-lysophosphatidic acid which labels sphingosine (S1P)-Rs; in subsequent experiments, binding to both ligands was abolished by short interfering RNA (siRNA) directed towards S1P_1_-Rs and S1P_3_-Rs (Molderings et al. 2007a). This finding is very interesting since the latter receptors and the LPA-Rs (the putative molecular entities of the presynaptic imidazoline receptors above) represent the two subgroups of the lysophospholipid receptors (Chun et al. 2010). Although ^3^H-idazoxan labels I_2_ sites both in the rat stomach (Molderings et al. 1998b) and bovine adrenal medulla (Molderings et al. 1994b), ^3^H-clonidine binds to different entities in either tissue. As opposed to I_1_ sites in the adrenal medulla, ^3^H-clonidine binds to σ-like receptors in the rat stomach (Molderings et al. 1995a), i.e. to receptors which were originally considered an opioid receptor subtype but later turned out to be two entities (σ_1_ and σ_2_) with entirely different pharmacological properties (reviewed in Kim and Pasternak 2017). Nonetheless, the σ-like receptors labeled with ^3^H-clonidine in the rat stomach are not identical with the σ_2_ sites labeled by ^3^H-1,2-di-(2-tolyl)guanidine in this tissue and were designated as non-I_1_-non-I_2_ sites instead (Molderings et al. 1998b). “True” σ_2_ sites, labeled by ^3^H-1,2-di-(2-tolyl)guanidine, could also be identified in human stomach and in N1E-115 neuroblastoma cells (Molderings et al. 1998b).

(ii) In several papers, efforts were made to define principal mechanistic properties of the imidazoline and σ_2_ sites. I_1_ binding (^3^H-clonidine; bovine adrenal medulla) was inhibited by the stable GTP analog 5′-guanylylimidodiphosphate (Gpp(NH)p), whereas binding to I_2_ (^3^H-idazoxan; bovine adrenal medulla) and non-I_1_-non-I_2_ sites (^3^H-clonidine; rat stomach) was not, suggesting that only I_1_ sites are G protein-coupled (Molderings et al. 1993, 1994b, 1995a). The coupling of I_1_ sites to G proteins was further supported by the finding that I_1_-Rs on PC12 cells can be classified as S1P-Rs (Molderings et al. 2007a), which belong to the G protein-coupled receptors (Chun et al. 2010). When S1P_3_-Rs (which most efficiently bind to G_q_ protein; Chun et al. 2010) were transiently expressed in human embryonic kidney (HEK) 293 cells, imidazolines consequently led to an increase in intracellular Ca^2+^ (Molderings et al. 2007a). For σ_2_ sites, an entirely different mechanism is very likely. Thus, imidazoline and σ ligands inhibited the 5-HT_3_-R-induced ^14^C-guanidinium influx in N1E-115 cells (a model of cation influx); the results suggest that this effect is, at least in part, related to an interaction with σ_2_ sites (Molderings et al. 1996b).

(iii) Finally, studies were carried out to determine functional effects associated with imidazoline and related receptors. Molderings et al. (2007b) found that ligands at imidazoline/S1P-Rs inhibited the protein content (which represents an estimate for cell numbers) in PC12 cells; this effect could be reduced by siRNA species directed towards S1P_1_-Rs, S1P_2_-Rs, and S1P_3_-Rs. Imidazolines and/or σ ligands did not affect the tone of rat gastric strips and acid secretion, suggesting that I_2_, non-I_1_-non-I_2_, or σ_2_ sites are not implicated in the latter two functional effects (Molderings et al. 1998b). Despite their lack of effect on acid secretion, imidazolines led to an increase in histamine release in the rat stomach (Molderings et al. 1999c), which, most probably, is related to the inhibition of K_ATP_ channels, i.e. the mechanism known for the increase in insulin release elicited by imidazolines (Chan 1998) or sulfonylureas (Rorsman et al. 1990). The facilitatory effect on histamine release was shared by agmatine which was identified in human gastric juice in high concentrations and showed an even higher concentration in the stomach of *Helicobacter pylori*-positive subjects, pointing to a pathophysiological role in gastroduodenal ulcer (Molderings et al. 1999a). Apart from the latter effect in the stomach, imidazolines were suggested to have various effects also on cardiovascular functions (Molderings and Göthert 1999). Endogenous ligands at imidazoline-Rs (and α-ARs) may play a role in vascular smooth muscle proliferation and blood pressure regulation and the imidazoline moxonidine and the oxazoline rilmenidine appear to exert their antihypertensive effect at least partially via I_1_-Rs (Schäfer et al. 1995). Exogenously added imidazolines may also possess antiarrhythmic effects implicating central and peripheral and α_2_-AR-dependent and α_2_-AR-independent sites of action (Molderings and Göthert 1999). They may, however, also interfere with the beneficial cardiac effects of compounds activating K_ATP_ channels due to their known antagonistic effect at this mechanism.

#### Agmatine

**Fig. 8:**
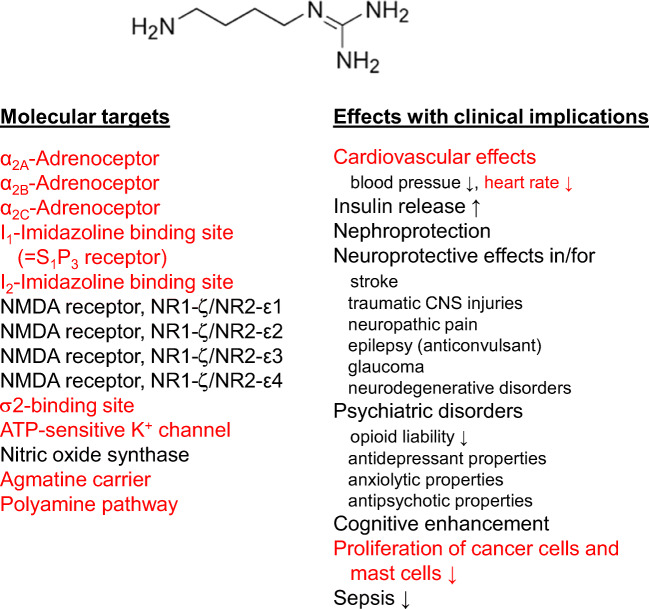
The many faces of agmatine. Aspects studied by Manfred Göthert are marked with red color

Agmatine, decarboxylated arginine, is an aminoguanidine that was discovered by the Nobel laureate A. Kossel (Kossel 1910) in bacteria and plants. More than 80 years later, Reis and coworkers purified agmatine from bovine brain and they discovered that agmatine is an endogenous ligand at imidazoline binding sites (Li et al. 1994). Thereafter, a variety of agmatine-mediated effects in mammals has been described (for recent reviews, see Laube and Bernstein 2017; Xu et al. 2018). In the late 1990s, Manfred Göthert and G.J. Molderings started to examine agmatine (Fig. 8). In a study on the expression of imidazoline binding sites in rat and human stomach, they demonstrated that *Helicobacter pylori* is able to form and to release the endogenous imidazoline-R ligand agmatine and that considerable amounts of agmatine are present in human gastric juice, especially in that from *H. pylori*-positive patients (Molderings et al. 1999a, d). Shortly thereafter, they showed that at the α_2D_-AR of rat vena cava and brain cortex (the α_2D_-AR is the species homolog of the α_2A_-AR) agmatine acts as a competitive antagonist at the ligand recognition site and that it enhances the effects of agonists probably by binding to an allosteric site which seems to be labeled by agmatine (Molderings et al. 2000b).

Since agmatine had been shown to be degraded in mammalian tissues not only to urea but also to the polyamine putrescine, Manfred Göthert examined whether both polyamines are taken up by the same or by different transport systems in the human glioma cell line SK-MG-1 (Molderings et al. 2001). This study demonstrated the existence of a specific uptake system for agmatine which is not identical with that for putrescine. In addition, agmatine uptake was not due to the activity of organic cation transporters such as OCT1, OCT2, OCT3, OCTN1, or OCTN2 neither in human SK-MG-1 glioma cells nor in six human intestinal tumor cells (Heinen et al. 2003; Molderings et al. 2003b). Since agmatine is present in human gastric juice, it was of interest to prove whether exogenous agmatine is taken up in the stomach. By in vitro exposure of rat isolated stomach to ^14^C-agmatine and by oral administration to rats in vivo, Manfred Göthert showed that this polyamine is accumulated in the stomach wall and distributed in various tissues and that the accumulation of agmatine was dose-dependently decreased by simultaneous administration of putrescine (Molderings et al. 2002b). The results indicated a transport system for agmatine and they were compatible with the idea of an entero-hepatic recirculation of agmatine (Molderings et al. 2002b; Fig. 8). In a further in vivo study with rats, administration of agmatine after partial hepatectomy was shown to reduce liver regeneration indicating a potential contribution of agmatine to the development of liver diseases (Molderings et al. 2003c). Agmatine, on the other hand, was shown to inhibit concentration-dependently the proliferation of six human intestinal tumor cell lines; in addition, the agmatine content in colon carcinoma tissue from patients who underwent surgery was much lower than in adjacent normal tissue which was interpreted to indicate an antineoplastic action of agmatine (Molderings et al. 2004). In a subsequent investigation, Manfred Göthert also demonstrated an antiproliferative effect of agmatine in rat and human hepatoma cells, indicating an involvement of agmatine in liver cell growth (Kribben et al. 2004). In a study in tumor cells of colonic, hepatic, and neuronal origin, Manfred Göthert examined the molecular basis for the antiproliferative effect of agmatine (Wolf et al. 2007). Agmatine inhibited the proliferation of all examined cells. At the human hepatoma cell line HepG2, it was demonstrated that the antiproliferative effect is due to an interaction with neither the NO synthases, the polyamine-dependent hypusination of the translation factor elF5a, nor an agmatine-induced reduction in availability of intracellular arginine but it may be due to an increase in intracellular caspase-3 activity, indicating a promotion of apoptosis (Wolf et al. 2007; Fig. 8). Finally, regulatory mechanisms underlying agmatine homeostasis in humans have been explored in human colon resectates by measuring mRNA encoding ornithine decarboxylase (ODC), diamine oxidase (DAO), and arginine decarboxylase (ADC), from the production of agmatine by 10 cultured bacterial strains of the residual intestinal microflora and from measurement of agmatine in portal venous blood plasma (Haenisch et al. 2008). The study showed that (i) the level of mRNA was lower (ADC and DAO) or higher (ODC) in neoplastic tissue than in the adjacent normal tissue, that (ii) bacteria strains considerably differed in agmatine production, and (iii) a substantial hepatic agmatine removal from blood occurred. Thus, a perturbation of agmatine homeostasis has been proven to be involved in the regulation of malignant cell proliferation, and agmatine available for intestinal absorption may differ considerably depending on the composition of the bacterial flora, and finally, the liver plays a crucial role in the maintenance of agmatine homeostasis in the human organism (Haenisch et al. 2008).

### Cannabinoids

**Fig. 9:**
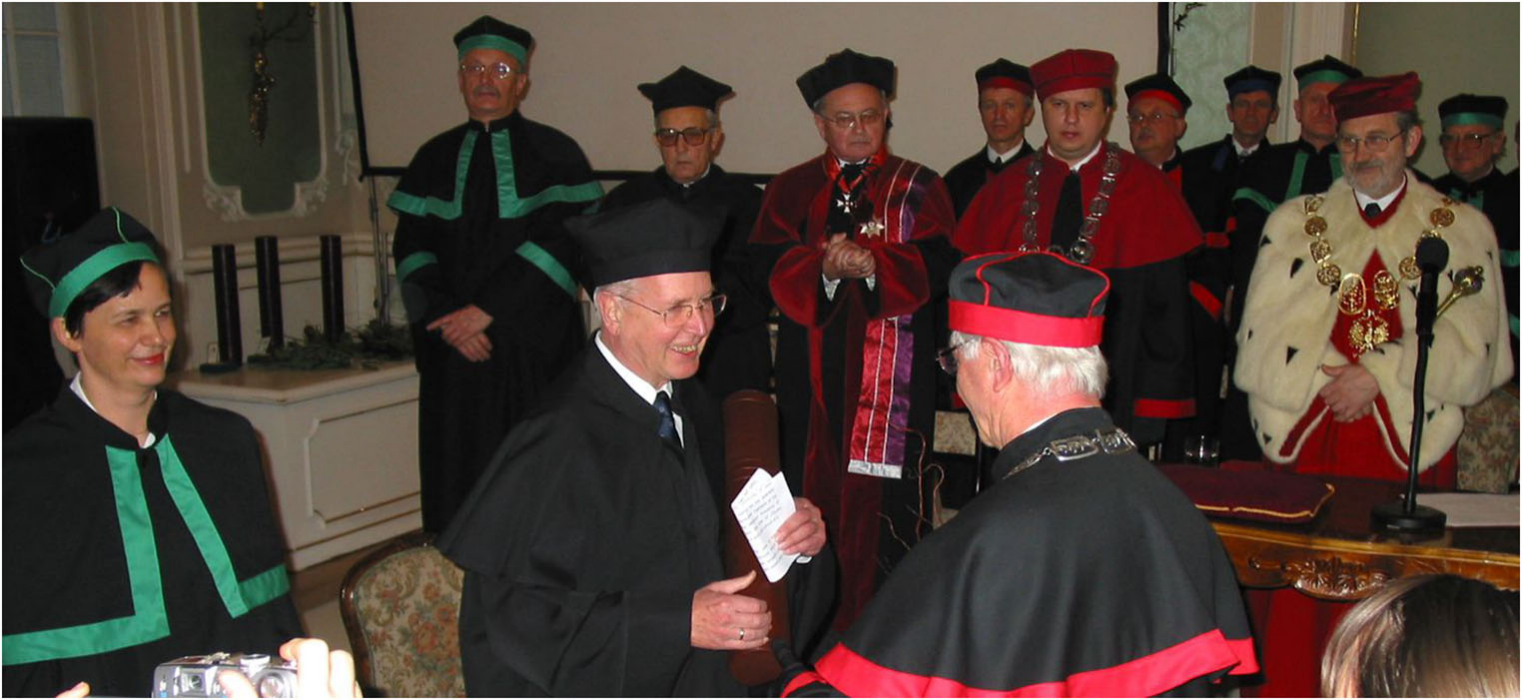
The ceremony of awarding the title of Doctor honoris causa of the Medical University of Białystok (Poland) to Manfred Göthert (12 December 2003). First line: Barbara Malinowska, Manfred Göthert, and Maciej Kaczmarski. Second line: Włodzimierz Buczko, Edmund Przegaliński, Zbigniew Herman, Jacek Nikliński, and Jan Górski. Note that B. Malinowska and W. Buczko (e.g., Malinowska et al. 1995) and E. Przegaliński (Przegaliński et al. 2005) cooperated with Manfred Göthert

Cannabinoids have been attracting the attention of Manfred Göthert since 1995. He became interested (i) in presynaptic CB_1_-Rs (and was supported in this respect by E. Schlicker, K. Fink, and G.J. Molderings), (ii) in cardiovascular effects of the endocannabinoid anandamide, and (iii) in CB_1_-R-independent effects of cannabinoids on 5-HT_3_-Rs and nACh-Rs (supported by B. Malinowska; see Fig. 9). Part of the latter studies was carried out in the laboratory of B. Malinowska when Manfred Göthert was awarded an Alexander von Humboldt Polish Honorary Research Fellowship after his retirement (2006). This fourth part of his professional career (following Hamburg, Essen, and Bonn) lasted from 2006 to 2009 and was split into several periods.

(i) CB_1_-Rs are typically located presynaptically on neurones and their activation leads to the inhibition of the release of the respective neurotransmitter (Schlicker and Kathmann 2001; Szabo and Schlicker 2005). Examples of such receptors could be identified on noradrenergic neurones of the hippocampus and of blood vessels (Table 4) and on the dopaminergic amacrine cells of the retina (Schlicker et al. 1996b). Presynaptic CB_1_-Rs were found in human and guinea pig but not rat and mouse hippocampus (Schlicker et al. 1997b). This finding stresses once again that one has to be careful when using rodent tissues as a model for humans. The CB_1_-R inverse agonist rimonabant facilitated (or tended to facilitate) transmitter release in the human and guinea-pig hippocampus (Schlicker et al. 1997b) and in the guinea-pig retina (Schlicker et al. 1996b). Although this phenomenon may be related to an increased formation of endocannabinoids (e.g., anandamide or 2-arachidonoylglycerol), the alternative explanation, i.e. that the CB_1_-Rs are constitutively active, is at least as likely (Pertwee 2005; Szabo and Schlicker 2005). It is of interest in this context that the density of CB_1_-Rs in some brain regions exceeds 1 pmol/mg and is higher than that of any other G protein-coupled receptor (Baker et al. 2003).

It is tempting to assume that the CB_1_-Rs identified in human hippocampus also contribute to the effects of hashish/marijuana on cognitive functions although one has to consider that presynaptic inhibitory CB_1_-Rs also occur on hippocampal *glutamatergic* neurones which prevail when compared to noradrenergic neurones (Szabo and Schlicker 2005). A similar reasoning may hold true for rimonabant, which was available from 2006 to 2009 as an anti-obesity agent and was withdrawn from the market due to its potential of serious psychiatric disorders (Ioannides-Demos et al. 2011). Provided that the retinal CB_1_-Rs also occur in humans, the possibility has to be considered that decreased retinal dopamine release is the biological substrate for the use of cannabis by Caribbean fishermen to ameliorate their night vision (West 1991). It has been shown for mice that the decrease in retinal dopamine occurring by night is associated with an increased rod electrical coupling (Jin et al. 2015). In that study, the rod coupling could be further increased by a D_2_-R antagonist and it would be plausible that the same may hold true for the inhibition of dopamine release.

(ii) Anandamide induces complex cardiovascular effects. In urethane-anesthetized mice and rats, rapid intravenous (i.v.) injection of anandamide elicits a triphasic response (e.g., Malinowska et al. 2001, 2010, 2012; Kwolek et al. 2005): phase I—a rapid, pronounced bradycardia and a transient drop in blood pressure; phase II—a brief pressor response, and phase III—a more prolonged, marked decrease in blood pressure. Since similar triphasic changes were also obtained after i.v. administration of methanandamide, a stable analog of anandamide, one can exclude the possibility that anandamide acts indirectly via its arachidonic acid metabolites (Malinowska et al. 2001). In conscious rodents, phase II is the most evident one, phase I is induced only by the higher doses of anandamide, and phase III is absent. As early as in 1996, it was shown that CB_1_-Rs are involved in the stimulation of phase III, since it was diminished by their antagonist rimonabant (for review, see Malinowska et al. 2012). Three years later, anandamide was recognized as endogenous ligand of vanilloid TRPV1-Rs, since both it and methanandamide were shown to induce vasodilation of rat isolated mesenteric and hepatic arteries and the guinea-pig basilar artery in a manner sensitive to the TRPV1-R antagonist capsazepine (for literature, see Malinowska et al. 2001). Phase I resembled the so-called Bezold-Jarisch reflex, which can be induced by the activation of TRPV1-Rs and 5-HT_3_-Rs located on vagal afferent C-fibers in the heart. Thus, M. Göthert decided to use this model in order to check whether anandamide activated TRPV1-Rs under in vivo conditions and we were the first demonstrating this property of anandamide. The working hypothesis of M. Göthert confirmed experiments in which the anandamide and/or methanandamide-induced phase I (i) was abolished by bilateral vagotomy and in pithed rats (the latter model offers the opportunity to study drug effects on the peripheral cardiovascular system only); (ii) was diminished by capsazepine and by the non-selective TRPV1-R antagonist ruthenium red but not by rimonabant; (iii) similarly to in vitro experiments, the TRPV1-R agonist capsaicin was more potent than anandamide and methanandamide in stimulation of phase I (Malinowska et al. 2001; Kwolek et al. 2005). Interesting enough, we found that acute myocardial ischemia enhances the vanilloid TRPV1-R-mediated Bezold-Jarisch reflex induced by low doses of anandamide (Lupiński et al. 2011).

M. Göthert had also decided to deal with mechanism(s) underlying phase II in urethane-anesthetized rats. Rimonabant and bilateral vagotomy failed to modify phase II excluding the involvement of CB_1_-Rs and the possibility that it was the simple response to the preceding hypotension. The additional use of pithed rats allowed us to determine peripheral and central components responsible for the anandamide-induced and/or methanandamide-induced phase II. The peripheral component (also observed in pithed rats; most probably located in blood vessels) was sensitive to nifedipine, ruthenium red, and pentobarbitone and, hence, probably represents a Ca^2+^-dependent mode of action. The central one (absent in pithed rats) was reduced by some β-AR antagonists (the non-selective propranolol and the β_2_-selective antagonist ICI118551 but not by the β_1_-AR antagonist CGP20712), by an NMDA-R (MK-801) and by thromboxane A_2_ (TP)-R antagonists (sulotroban, daltroban, and SQ 29548). In addition, all above compounds decreased the pressor response to intracerebroventricular (i.c.v.) injection of anandamide (studied in the presence of CB_1_-R and TRPV1-R antagonists). Anandamide and methanandamide failed to bind to TP-Rs on washed rat platelets and an inhibitor of thromboxane A_2_ synthase furegrelate i.c.v. reduced the pressor effect of anandamide i.v. suggesting that anandamide causes an increase in thromboxane A_2_ synthesis in the brain (Kwolek et al. 2005; Malinowska et al. 2010). We later identified the paraventricular nucleus as the possible central site of anandamide action (Grzęda et al. 2017).

(iii) The pioneering electrophysiological experiments on rat nodose ganglion neurones demonstrating that cannabinoid receptor agonists inhibited the 5-HT_3_-R-mediated currents (Fan 1995) allowed M. Göthert again to study the function of his favorite 5-HT_3_-Rs, this time in connection with cannabinoid pharmacology. Indeed, non-competitive inhibitory effects of cannabinoid-R agonists, mainly anandamide, CP55940, WIN55212-2 (but not its inactive S-(-)-enantiomer WIN55212-3), which were resistant to the CB_1_-R antagonist rimonabant, were determined in vitro on the 5-HT-induced current in HEK 293 cells expressing recombinant human 5-HT_3A_-Rs (Barann et al. 2002) and in vivo on the Bezold-Jarisch reflex induced by the 5-HT_3_-R agonist phenylbiguanide (but not by the TRPV1-R antagonist capsaicin) in urethane-anaesthetized rats (Godlewski et al. 2003; Table 2). The cannabinoids act probably at an allosteric modulatory site of the 5-HT_3_-R itself because of (i) the slow development of the inhibition (about 3 min in vitro and 10-20 min in vivo); (ii) the failure of cannabinoids to inhibit binding of the 5-HT_3_-R radioligand ^3^H-GR65630 to membranes of HEK 293 cells stably transfected with human 5-HT_3A_-Rs; and (iii) the lack of an inhibition of the 5-HT-induced current when the cannabinoids were administered to the patches exclusively during, but not before, stimulation with 5-HT. The necessity of stimulation of the 5-HT_3_-Rs at their orthosteric site for the inhibitory effect of anandamide exerted via its allosteric binding site was confirmed in double CB_1_/CB_2_-R knockout mice, in which the anandamide-induced analgesia (but not catalepsy) was reduced in the presence of the 5-HT_3_-R antagonist ondansetron preventing 5-HT tonically released from the adjacent serotoninergic nerve terminals from binding to the orthosteric site (Rácz et al. 2008). The inhibitory influence of cannabinoids on 5-HT_3_-Rs may be important in 5-HT_3_-R-mediated responses like analgesia and emesis.

One should keep in mind that the function of 5-HT_3_-Rs is modulated by numerous substances (e.g., Al Kury et al. 2018). We found that (+)-tubocurarine (but not another non-depolarizing neuromuscular blocking agent, pipecuronium) inhibited and substance P (but not mastoparan, a peptide from wasp venom that shares the property of substance P to activate G proteins) potentiated the 5-HT_3_-R (but not the TRPV1-R)-mediated Bezold-Jarisch reflex (Malinowska et al. 1996).

Anandamide and methanandamide allosterically inhibit the nicotine-evoked currents through recombinant homopentameric α7 nACh-Rs in Xenopus oocytes (for literature, see Baranowska et al. 2008). Allosteric sites on a transmitter-gated ion channel may be considered potential targets of new classes of therapeutic drugs. Thus, Manfred Göthert decided to check whether methanandamide inhibits the function of the above receptors under in vivo conditions in urethane-anesthetized pithed rats treated with atropine. Urethane anesthesia makes the potential involvement of presynaptic CB_1_-Rs unlikely (Kurz et al. 2009). We found that methanandamide (similarly to the subunit-non-selective nACh-R antagonist hexamethonium and the selective α7 nACh-R antagonist methyllycaconitine) reduced the nicotine-induced tachycardia (maximally by 40% in each case). Non-additivity of their inhibitory effects suggested that methanandamide acts probably at an allosteric site of α7 subunit-containing nACh-Rs (Baranowska et al. 2008).

## Conclusions

Manfred Göthert has published no less than 271 papers, 110 of which are related to serotonin; his h index amounts to 57 (Google Scholar, accessed on March 26, 2021). Some of his major scientific achievements and their clinical implications will be heralded below (“Major scientific achievements” and “Clinical implications” sections).

### Major scientific achievements

**Fig. 10:**
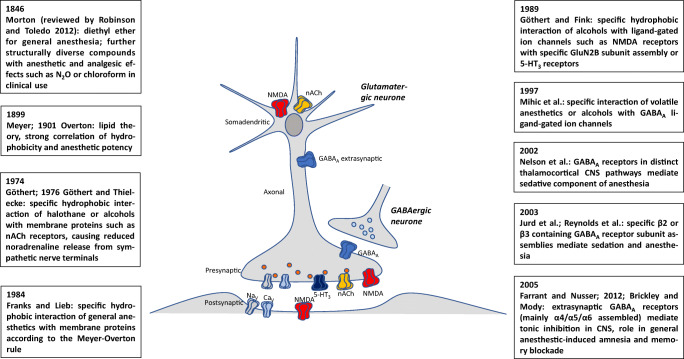
Milestones in the elucidation of the mode of action of general anesthetics and Manfred Göthert’s contributions (seminal papers are given in the boxes). Manfred Göthert showed that the *general anesthetic halothane* is a negative allosteric modulator at periperal nACh und 5-HT_3_ receptors; in other words, anesthetics have a much more specific effect than suggested by the Meyer-Overton hypothesis. According to Franks and Lieb (1984, 1997), anesthetics inhibit nACh and 5-HT_3_ receptors which are also present in the brain (see figure) and NMDA receptors solely occurring in the brain (see figure) and stimulate central GABA_A_ receptors (see figure). All receptors are ligand-gated ion channels; only the GABA_A_ receptors (today believed to be the major target of anesthetic action) are inhibitory. Manfred Göthert also showed that *ethanol* inhibits peripheral nACh and 5-HT_3_ receptors at concentrations obtained under moderate intoxication. Simultaneously with, but independent from, Lovinger et al. (1989), he found that ethanol inhibits NMDA receptors at concentrations occurring under social drinking. Note that the action of general anesthetics and ethanol is very selective: E.g., voltage-dependent cation channels (Na_V_, Ca_V_; see figure) are affected at extremely high concentrations only

The *mechanism of action of general anesthetics* was unclear over a long time period. The Meyer-Overton rule (1899-1901) only describes that there is an excellent correlation between their potency as anesthetics and their hydrophobicity (Fig. 10). There was general belief that the anesthetics act via unspecific hydrophobic interactions with membrane lipids or lipoproteins. By contrast, Manfred Göthert showed in 1974 that anesthetics are negative allosteric modulators of nACh-Rs (later extended to 5-HT_3_-Rs, another group of ligand-gated cation channels), i.e. that they exhibit specific hydrophobic properties. Using electrophysiological techniques, Franks and Lieb (1984) showed that anesthetics act on central NMDA-Rs, a third type of ligand-gated cation channels (Fig. 10). Subsequent studies revealed that they also act as positive allosteric modulators at GABA_A_-Rs, another example of ligand-gated ion channels which, however, lead to hyperpolarization as opposed to depolarization obtained with nACh-Rs, 5-HT_3_-Rs, and NMDA-Rs. Today, the concept of a specific interaction of anesthetics with GABA_A_-Rs is widely acknowledged as their site of action (Fig. 10). Unfortunately, the pioneering work of Manfred Göthert was not appreciated by the scientific community and, deeply disappointed, he wrote in retrospect (Göthert 2014): Thus, we were the first to identify the nACh and the 5-HT_3_ receptor, two ligand-gated ion channels, as sites of action of halothane. These results were obtained about one decade earlier than the same conclusions from electrophysiological data. My biochemical models obviously were unknown systems used by an unknown author, who published so far unknown results – a typical constellation for not being cited in the relevant literature.

Manfred Göthert also observed that *ethanol*, like the general anesthetics, inhibits peripheral nACh-Rs and 5-HT_3_-Rs and that the effect on the nACh-Rs occurred at ethanol concentrations compatible with moderate intoxication (Göthert and Thielecke 1976). More than 10 years later, he examined the effect of ethanol also on central NMDA-Rs (Fig. 10). Again, an inhibitory effect could be shown and the potency of ethanol at NMDA-Rs was even higher than that at peripheral nACh-Rs (Göthert and Fink 1989; see Fig. 5 in Göthert et al. 2020). The study by Göthert and Fink et al. (1989), together with that by Lovinger et al. (1989) based on an electrophysiological technique, demonstrates that the NMDA-R is a major site of action of ethanol.

For the sake of comparison, Manfred Göthert also considered noradrenaline release evoked by activation of G_q_ protein-coupled receptors, K^+^ depolarization, and electrical stimulation (Table 3). The fact that noradrenaline release evoked by the latter methods was affected by very high concentrations of halothane and ethanol only (if at all) shows again that the two compounds possess a highly specific mode of action. Electrical stimulation, via activation of voltage-dependent Na^+^ channels, eventually leads to opening of *voltage-dependent Ca*^*2+*^ *channels* (VDCC). It was tempting to assume that the VDCCs in the rabbit heart are blocked by the classical Ca^2+^ antagonists like verapamil but this held true for extremely high concentrations only, excluding that L-type VDCCs (present nomenclature: Ca_V_1.x) are involved (Göthert et al. 1979c). In subsequent years when more appropriate drug tools had become available, Manfred Göthert could show that the VDCCs in the rat and human neocortex belong to the P-/Q-type (Ca_V_2.1; major part) and N-type (Ca_V_2.2; minor part) (see the “Gabapentinoids and voltage-gated Ca^2+^ channels” section), whereas the VDCCs in the human atrium belong to the N-type (Ca_V_2.2; Molderings et al. 2000c). The L-type, P-/Q-type, and N-type VDCCs differ in their α_1_ subunit; another part of the VDCCs, the α_2_δ subunit, is inhibited by the gabapentinoids and Manfred Göthert could disclose a chain of events involved in their therapeutic action in rat and human brain (see the “Clinical implications” section).

**Fig. 11:**
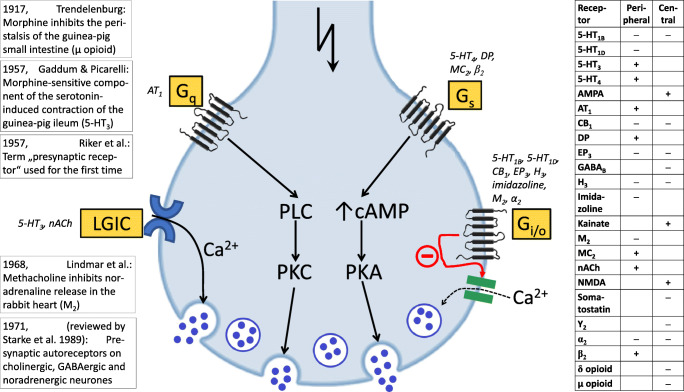
Milestones in the identification of presynaptic receptors and contributions of Manfred Göthert (seminal papers are given in the boxes). The figure shows that he studied the modulation of noradrenaline release from *postganglionic sympathetic neurones* by 15 types of presynaptic receptors. Activation of ligand-gated ion channels (LGICs), G_q_-coupled and G_s_-coupled receptors increases noradrenaline release (+; see vesicles fusing with the cell membrane and releasing noradrenaline molecules into the synaptic cleft); activation of G_i/o_ protein-coupled receptors decreases noradrenaline release (−). Signaling following activation of G protein-coupled receptors as described by Kubista and Boehm (2006). The types of presynaptic receptors studied by Manfred Göthert are given next to the yellow boxes; in the case of the 5-HT_4_-R, a parasympathetic neurone is interpolated and the increased release of ACh eventually leads to inhibition of noradrenaline release (for details, see the “Presynaptic serotonin heteroreceptors on noradrenergic neurones” section). On *noradrenergic and serotoninergic neurones of the brain*, only LGICs and G_i/o_ protein-coupled presynaptic receptors occur (not shown) and 13 types of presynaptic receptors were identified by Manfred Göthert (see table on the right hand side). Altogether, 23 different types of presynaptic receptors were examined. *ACh*, acetylcholine; *PKA*, protein kinase A; *PKC*, protein kinase C; *PLC*, phospholipase C

Part of the nACh-Rs, 5-HT_3_-Rs, and NMDA-Rs mentioned above are presynaptic receptors which represent another research field in which Manfred Göthert excelled. Examples of *presynaptic receptors* have been described decades ago but systematic studies started around 1970 only (Fig. 11). Manfred Göthert became interested in this topic from 1974 on and in the subsequent three decades, there was a lively competition between the groups of E. Muscholl in Mainz, K. Starke in Freiburg, and Manfred Göthert in Essen and since 1986 in Bonn; many of the numerous papers dedicated to this topic appeared in *Naunyn-Schmiedeberg’s Arch Pharmacol*. Manfred Göthert studied 23 different types of presynaptic receptors (Fig. 11); he was most interested in presynaptic receptors on central serotoninergic and noradrenergic neurones and on peripheral noradrenergic neurones. He was the first to identify presynaptic somatostatin and ACTH (MC_2_)-Rs (Göthert 1980b, 1981) and (together with Cerrito and Raiteri 1979) serotonin autoreceptors (Göthert and Weinheimer 1979). Twelve receptors were also examined in human tissues (Tables 1 and 4). Although the mere identification of presynaptic receptors was important per se (since this area was terra incognita at that time), Manfred Göthert provided more than just functional anatomy. Thus, his studies were helpful with respect to the elucidation of receptor classification (e.g., of serotonin receptor subtypes, see next paragraph). Moreover, he disclosed the inhibitory interaction of different types of G_i/o_ protein-coupled presynaptic receptor types with each other or revealed the mechanisms behind the receptor level (e.g., by increasing cAMP levels or inhibiting G_i/o_ protein by N-ethylmaleimide or pertussis toxin).

Manfred Göthert is identified with *serotonin* by many colleagues and indeed research dedicated to this monoamine accompanied him from the early seventies of the previous century until his death. When his interest for serotonin was kindled for the first time, there was only the simple classification of Gaddum and Picarelli (1957), encompassing D (5-HT_2A_)-Rs and M (5-HT_3_)-Rs. Using organ bath studies and experiments on pithed rats and on anesthetized animals, he identified new models of those receptors or refined the available ones. Moreover, he showed effects of serotonin itself or of its derivatives on postsynaptic α-adrenoceptors or examined their indirect sympathomimetic effect. In cooperation with H.G. Baumgarten, he studied the selectivity of the neurotoxins 5,6-DHT and 5,7-DHT in terms of serotoninergic and noradrenergic neurones (“Early studies” section). Few years later, a new serotonin receptor classification (5-HT_1_ and 5-HT_2_), based on radioligand binding studies, was proposed by Peroutka and Snyder (1979). Manfred Göthert also switched to methods based on radioligands, i.e. superfusion studies on tissues preincubated with ^3^H-serotonin, and identified presynaptic serotonin autoreceptors and heteroreceptors as well as presynaptic heteroreceptors on serotoninergic neurones (“Presynaptic serotonin heteroreceptors on noradrenergic neurones” and “Presynaptic heteroreceptors on serotoninergic neurones” sections, Tables 1 and 2). Again some years later, serotonin receptor subclassification was no longer based on native but on cloned receptors (starting with Lübbert et al. 1987). Manfred Göthert took the opportunity to further refine his methodological armamentarium and compared the properties of naturally occurring mutants of human serotonin receptors with their respective wild types both of which were expressed in cell lines. Differences were obtained for almost all receptors under study and at least some of them are clinically relevant (see the “Clinical implications” section).

**Fig. 12:**
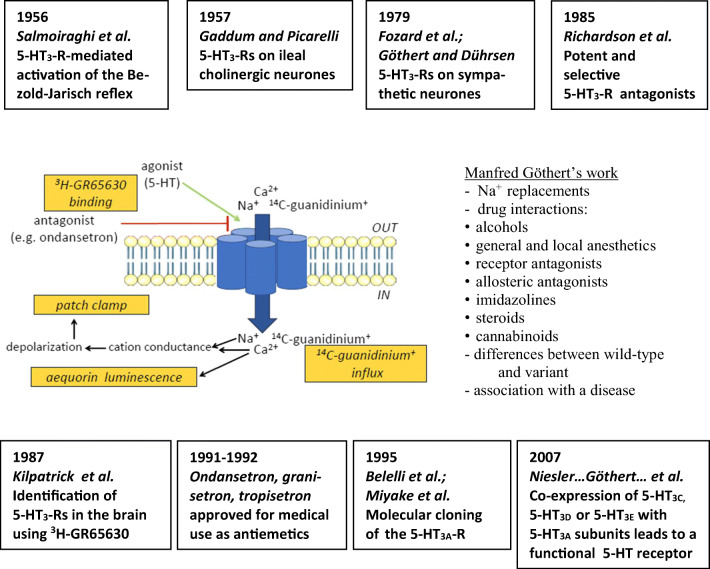
Milestones in determination of 5-HT_3_-R structure and function and some methods and results of the work of Manfred Göthert (seminal papers are given in the boxes)

When Manfred Göthert was retired in 2006, the number of serotonin receptor families had increased to seven and the complete number of subtypes had reached at least 14 entities (Göthert et al. 2020). As a matter of fact, he dealt with 6 of the 7 receptor families (except for 5-HT_6_-Rs) but *5-HT*_*3*_*-Rs* fascinated him most (Fig. 12). Three aspects will be briefly discussed. First, simultaneously with, but independently from, Fozard et al. (1979), he identified the 5-HT_3_-R leading to noradrenaline release in the rabbit heart; this presynaptic receptor serves as one of the peripheral targets of anesthetics (Tables 2 and 3). Second, he provided an in-depth analysis of the human 5-HT_3_-R expressed in a cell line, using four elegant methods (Table 2, Fig. 12). Third, he studied splice variants and naturally occurring variants of this receptor and was involved in the delineation of the 5-HT_3C_, 5-HT_3D_, and 5-HT_3E_ subtypes (Figs. 4 and 12).

### Clinical implications

The oeuvre of Manfred Göthert not only is important from the viewpoint of science but also has numerous clinical implications some of which will be highlighted here.

- His discovery that the inhibitory effect of *halothane* on nACh-R-mediated noradrenaline release in cardiovascular tissues occurs in a concentration range obtained under general anesthesia was an early hint that this anesthetic has a much more specific site of action than believed before (Göthert 1974).
- The inhibitory effect of *ethanol* on NMDA-R-mediated transmitter release in the brain represents one of its major sites of action (Göthert and Fink 1989).
- The inhibitory effect of *gabapentin and pregabalin* (inhibitors of the α_2_δ subunit of voltage-dependent Ca^2+^ channels) on AMPA-induced noradrenaline release occurred under clinically relevant concentrations of these drugs (Fink et al. 2002b).

Manfred Göthert has identified numerous sites of presynaptic receptors involved in the main action or the side effects of drugs.

- The serotonin autoreceptor in the brain may serve as an example. Many *antidepressants*, including the selective serotonin re-uptake inhibitors (SSRIs), have an indirect effect on the autoreceptor, i.e. the increased serotonin concentration in the synaptic cleft downregulates the density of the autoreceptor via which the monoamine restricts its own release; as a consequence, serotonin release is gradually increasing. This phenomenon explains why the effect of such antidepressants is developing with a time lag of some days or few weeks (Göthert and Schlicker 1993). Although it is an attractive hypothesis that 5-HT_1B_-R antagonists, by interrupting the negative feedback loop, may elicit an immediate increase in serotonin release associated with an instantaneous onset of antidepressant activity, little evidence for such antidepressants is currently available (Tiger et al. 2018). Nonetheless, interesting data exist for the β-blocker pindolol, which also blocks presynaptic 5-HT_1B_ autoreceptors and, at even lower concentrations, somadendritic 5-HT_1A_ autoreceptors. In some but not all clinical studies, pindolol accelerated and/or increased the antidepressant activity of SSRIs when given in combination (Artigas et al. 2018).

Manfred Göthert studied the molecular properties of naturally occurring serotonin-R-variants in cell lines transfected with the respective cDNAs. Two examples for which differences between mutant and wild type exist and additional studies in humans or in human tissue were performed will be heralded here.

- First, the *Arg219Leu variant of the 5-HT*_*1A*_*-R* is associated with major depression and may play a role in the pathogenesis of depression (Haenisch et al. 2009).
- Second, occurrence of the *Phe124Cys variant of the 5-HT*_*1B*_*-R* led to a more marked contraction of human vessels to serotonin and may explain the increased liability of some individuals to sumatriptan-induced vasospasm (Verheggen et al. 2006).

The aforementioned examples are related to ligand-gated and voltage-dependent cation channels, to presynaptic receptors and to serotonin receptors, the classical topics of Manfred Göthert, but one should not overlook important clinical implications from another two areas of his research.

- At the very beginning of his scientific career, Manfred Göthert dealt with *carbon monoxide* toxicology. His studies revealed that lowering of the general threshold limit value (MAK) for CO from 100 to 50 ppm was justified but that a special adjustment for Caisson workers was not necessary (“Carbon monoxide toxicology” section).
- Towards the end of his career, Manfred Göthert became interested in *agmatine*. He was involved in studies in which agmatine formation by bacteria in the human gastrointestinal tract, its handling by the human body (including a special carrier), and its putative antiproliferative effect in human tissue was described (Fig. 8).

### Personal remarks

Aspects apart from scientific issues have not been considered here. Thus, Manfred Göthert served as highly estimated academic teacher or as dedicated Dean of the Medical Faculty of the University of Bonn (1998-2002) and President of the German Society for Experimental and Clinical Pharmacology and Toxicology (DGPT; 1997-1999) and the Federation of European Pharmacological Societies (EPHAR; 2004-2006). Manfred Göthert excelled through all aspects of his professional career. We remember him “as a constantly smiling man, an eternal optimist, determined in his views, kind, straightforward and spontaneous in dealing with other people, who did not care about maintaining a distance between him as a boss and colleagues and for whom being a scientist was not only a profession but a lifestyle” (obituary by Malinowska et al. 2020). We dearly miss him.

## Abbreviations

2-Methyl-5-HT: 2-Methyl-5-hydroxytryptamine
5-CT: 5-Carboxamidotryptamine
5-HT: 5-Hydroxytryptamine (serotonin)
5,6-DHT: 5,6-Dihydroxytryptamine
5,7-DHT: 5,7-Dihydroxytryptamine
6-HT: 6-Hydroxytryptamine
8-OH-DPAT: 8-Hydroxy-2-(di-n-propylamino)tetralin
ACTH: Adrenocorticotropic hormone
ACh: Acetylcholine
ADC: Arginine decarboxylase
AMPA: α-Amino-3-hydroxy-5-methyl-4-isoxazolepropionic acid
AR: Adrenoceptor
COS: Cells being ***C***V-1 (simian) in ***o***rigin, and carrying the ***S***V40 genetic material
CRC: Concentration-response curve
DAO: Diamine oxidase
edg: Endothelial differentiation gene
GABA: γ-Aminobutyric acid
GppNHp: Guanosine-5′-[β,γ-imido]triphosphate
GTPγS: Guanosine-5′-[γ-thio]triphosphate
HEK: Human embryonic kidney
i.v.: Intravenous
LGIC: Ligand-gated ion channel
LPA: Lysophosphatidic acid
MAC: Minimal alveolar concentration
mACh: Muscarinic acetylcholine
MAK: Maximale Arbeitsplatzkonzentration (maximum concentration in the work place)
nACh: Nicotinic acetylcholine
NMDA: N-Methyl-D-aspartate
NMDG: N-Methyl-D-glucamine
OCT, OCTN: Organic cation transporters
ODC: Ornithine decarboxylase
PC12: Pheochromocytoma 12
ppm: Parts per million
R: Receptor
SEM: Standard error of the mean
SHR: Spontaneously hypertensive rat
siRNA: Short interfering RNA
SNP: Single-nucleotide polymorphism
SSRI: Selective serotonin re-uptake inhibitor
TM: Transmembrane domain
TMA: Tetramethylammonium
Tris: Tris(hydroxymethyl)aminomethane
TRPV: Transient receptor potential vanilloid
UTR: Untranslated region
VDCC: Voltage-dependent calcium channels
τ_OFF_: Inactivation time constant
τ_ON_: Onset time constant
